# Analysis of microbiome high-dimensional experimental design data using generalized linear models and ANOVA simultaneous component analysis

**DOI:** 10.3389/frmbi.2025.1584516

**Published:** 2025-10-15

**Authors:** Fentaw Abegaz, Davar Abedini, Lemeng Dong, Johan A. Westerhuis, Fred van Eeuwijk, Harro Bouwmeester, Age K. Smilde

**Affiliations:** ^1^ Swammerdam Institute for Life Sciences, University of Amsterdam, Amsterdam, Netherlands; ^2^ Biometris, Wageningen University Research, Wageningen, Netherlands

**Keywords:** generalized linear models, ANOVA simultaneous component analysis, experimental design, high dimensional microbiome data, differential abundance analysis, Tweedie model

## Abstract

In microbiome studies, addressing the unique characteristics of sequence data—such as compositionality, zero inflation, overdispersion, high dimensionality, and non-normality—is crucial for accurate analysis. In addition, integrating experimental design elements into microbiome data analysis is important for understanding how factors such as treatment, time, and interactions affect microbial abundance. To achieve these objectives, we developed a new method that combines generalized linear models (GLMs) with ANOVA simultaneous component analysis (ASCA), which we term GLM-ASCA. This method aims to improve microbiome analysis by providing a more comprehensive understanding of differential abundance patterns in response to experimental conditions. GLM-ASCA models the unique characteristics of microbiome sequence data with GLMs and uses ASCA to effectively separate the effects of different experimental factors on microbial abundance. We evaluated GLM-ASCA using simulated data and subsequently applied it to real data to analyze the effect of nitrogen deficiency on root microbiome recruitment in tomato. Simulation studies demonstrated the effectiveness of GLM-ASCA in analyzing microbiome data in complex experimental designs, and the real-data application revealed valuable insights into the dynamics of microbial communities under nitrogen starvation, including the identification of beneficial bacterial species that promote tomato (*Solanum lycopersicum*) growth and health through nitrogen fixation.

## 1 Introduction

In microbiome research, high-throughput sequencing techniques such as amplicon sequencing (e.g., 16S rRNA gene sequencing) and whole-genome shotgun sequencing have become standard approaches for generating data from samples obtained from well-designed experiments aimed at understanding the mechanisms governing host–microbiome interactions (Zancarini et al., 2021). The microbiome count data produced through these sequencing methods typically exhibit distinct characteristics, including compositionality, zero inflation, overdispersion, high dimensionality, and non-normality. Various statistical tools have been developed to analyze microbiome data while addressing one or more of these characteristics, such as MaAsLin2: Multivariable Association with Linear Models 2 (Mallick et al., 2021) and LinDA: Linear Models for Differential Abundance (Zhou et al., 2022). While these tools implement univariate generalized linear models (GLMs) and are effective in identifying associations between individual features and covariates, they are limited in capturing the multivariate structure of the data or the joint effects of multiple factors across features.

It is also worthwhile to integrate treatment designs into statistical models to effectively address relevant research questions (Smilde et al., 2005). This facilitates precise accounting of the details of the treatment design, such as intervention time, treatment variations, and interactions between multiple factors, thereby allowing for an accurate assessment of how each factor and their combinations affect microbial abundance. However, in plant microbiome studies involving hundreds to thousands of correlated features and a limited number of samples, addressing experimental design elements such as treatment, time, and interactions, along with analyzing the specific characteristics of microbiome data, remains a considerable challenge.

In this context, several developments have incorporated techniques such as ANOVA-based partitioning of sources of variation using multivariate methods, with the aim of accounting for both the inherent data characteristics and the study design. One prominent method in this regard is ANOVA simultaneous component analysis (ASCA/ASCA+) (Smilde et al., 2005; Jansen et al., 2005; Thiel et al., 2017; Bertinetto et al., 2020; Martin and Govaerts, 2020; Thiel et al., 2023). ASCA/ASCA+ combines dimension reduction projection techniques with traditional linear statistical modeling to identify the main sources of variability in the resulting responses. It also provides visually interpretable representations of factor effects and their interactions, facilitating the interpretation of multivariate structures within the statistical model related to the experimental design (Smilde et al., 2005; Thiel et al., 2017). Moreover, ASCA/ASCA+ has been modified to cope with multivariate data in unbalanced multifactorial designs using weighted-effect ASCA (WE-ASCA) (Ali et al., 2020). ASCA+ has also been extended to analyze longitudinal data using linear mixed-effects models (Martin and Govaerts, 2020; Madssen et al., 2021; Jarmund et al., 2022). Furthermore, variable selection approaches have been implemented in VASCA (variable-selection ASCA) (Camacho et al., 2023) using permutation-based testing, and in GASCA (group-wise ASCA) (Saccenti et al., 2018) utilizing sparse group-wise principal component analysis (PCA).

Multivariate methodologies such as ASCA have proven useful in the analysis of metabolomics data, where linear models and PCA can be reasonably applied to continuous data. In the case of microbiome studies, however, the ASCA+ framework needs to be adapted to account for nonlinear and non-normally distributed data. In particular, extending the linear modeling approach in ASCA to GLMs, which assume an exponential family of probability distributions to accommodate data features such as counts, zero inflation, and overdispersion, would greatly enhance its applicability to microbiome data. In this work, we introduce GLM-ASCA (generalized linear models–ANOVA simultaneous component analysis), a novel approach for the analysis of microbiome data. GLM-ASCA incorporates the experimental design within a multivariate framework, providing a stronger statistical foundation for addressing the complexities of microbiome data analysis.

In linear models that employ ordinary least squares, orthogonal effect decomposition with ASCA/ASCA+ enables the separation and identification of distinct sources of variation in multivariate datasets, allowing for more precise and insightful analysis, particularly for data resulting from balanced experimental designs. In contrast, in GLMs, the iterative reweighted least squares (IRLS) algorithm is widely used to find maximum likelihood estimates rather than the ordinary least squares algorithm applied in linear models. As a result, parameter estimation in GLMs heavily relies on observation weights determined by the specific exponential family model under consideration. Including observation weights in GLMs complicates orthogonal effect decomposition, regardless of whether the treatment design is balanced. For this reason, it is crucial to appropriately extend ASCA within the framework of GLMs. In this context, we introduce methods for achieving orthogonal effect decomposition in GLMs for balanced design data to effectively utilize ASCA when integrated with GLMs. Thus, GLM-ASCA provides distinct advantages in well-structured experimental designs (e.g., full factorial designs, repeated measures) by decomposing variation attributable to main effects and interactions while accounting for the underlying multivariate structure. This integrated approach enables more transparent interpretation, improves the detection of biologically meaningful effects that may be missed by univariate methods, and enhances the identification of key features driving differential responses to experimental conditions.

To evaluate the performance of GLM-ASCA, we conducted a simulation study, which revealed that GLM-ASCA performs well, particularly in small-sample settings, motivating its application to microbiome experimental data. Subsequently, we applied GLM-ASCA to real microbiome data from tomato plants subjected to nitrogen starvation over time. Nitrogen is essential for plant fitness and productivity; however, non-legume crop plants mainly rely on chemical fertilizers. The application of these chemicals has severe environmental consequences, including water pollution from nutrient runoff, disruption of aquatic ecosystems through eutrophication, and the release of greenhouse gases that contribute to climate change (Savci, 2012). Identifying beneficial microorganisms capable of fixing atmospheric nitrogen (*N*_2_), reducing denitrification, and releasing inorganic nitrogen through mineralization—as well as the chemical communication that plants use to recruit them—can reduce the use of chemical fertilizers and pave the way toward sustainable agriculture and healthy ecosystems (Moreau et al., 2019; Mahmud et al., 2020; Abedini et al., 2021).

The paper is organized as follows: Section 2 introduces GLM-ASCA and demonstrates orthogonal decomposition in GLMs. Section 3 presents the performance of GLM-ASCA on simulated microbiome datasets and provides the analysis of plant microbiome data. Finally, Section 4 concludes the work with a discussion of the main results and future directions.

## 2 Methods and materials

### 2.1 GLM-ASCA

#### 2.1.1 GLM-ASCA decomposition

Consider a model containing *F* main and interaction effects so that the design matrix **X** can be decomposed into *F* + 1 blocks: **X** = (*X*_0_,**X**_1_ *,…*,**X** *_F_*), where **X** *_f_* is a matrix depending on the levels of factor *f* and *X*_0_ is a column vector of ones to estimate the intercept. Let **Y** = (**y**_1_ *,…*,**y** *_p_*) represent the *n* × *p*-dimensional response matrix. In order to accommodate various types of response data, including continuous, count, binary, and categorical variables, we extend the standard ASCA framework to be applied with Generalized Linear Models. That is, unlike ASCA, which uses ANOVA or linear regression, GLM-ASCA utilizes appropriate Generalized Linear Models to each column of the multivariate response matrix **Y**. In particular, GLM-ASCA decomposes the working response matrix rather than the observed response matrix **Y** consistent with the standard extension of LMs to GLMs (Lovison, 2014).

In GLM-ASCA, univariate GLMs are first fitted to each column of the multivariate response matrix **Y** = (**y**_1_ *,…*,**y** *_p_*) using the same design matrix **X**. Consider the *j*-th response variable **y** *_j_* = (*y_j_*_1_ *,…,y_jn_*), representing a vector of *n* observations with mean *µ_j_*. *A* GLM is specified for **y_j_** with linear predictor *η_j_* = **X** *β_j_*, link function *g*(*µ_j_*) = *η_j_*, and variance function *V* (***µ_j_***) = diag(*V* (*µ_ij_*)). The maximum likelihood estimate of the regression coefficients (*β_j_*) is given by (McCullagh, 2019):


$${\hat{\beta }}_{j}={\left(X^{T}{\hat{W}}_{j}X\right)}^{-1}X^{T}{\hat{W}}_{j}{\hat{z}}_{j},$$

where ${\hat{z}}_{j}={\hat{\eta }}_{j}+{\hat{r}}_{j}^{w}$ is the working response, with ${\hat{\eta }}_{j}=X{\hat{\beta }}_{j}$ , and ${\hat{r}}_{j}^{w}={\hat{D}}_{j}^{-1}(y_{j}-{\hat{\mu }}_{j})$ the working residuals. Here, ${\hat{D}}_{j}$ is a diagonal matrix with entries $(\frac{\partial \mu_{ij}}{\partial \eta_{ij}}){|}_{{\hat{\eta }}_{ij}}$ , i=1,…,n , and ${\hat{W}}_{j}$ is a diagonal matrix of weights with elements ${\hat{w}}_{ij}={\left(\frac{\partial \mu_{ij}}{\partial \eta_{ij}}{|}_{{\hat{\eta }}_{ij}}\right)}^{2}/V({\hat{\mu }}_{ij})$ . Details on GLMs are given in the **Supplementary** **Material Text S1** and **Supplementary Table S1**.

For all p responses in Y , the estimated parameter vectors ${\hat{\beta }}_{j}$ are collected as columns of the matrix $\hat{B}$ :


(1) $$\hat{B}=(\begin{array}{cccc} {\hat{\beta }}_{01} & {\hat{\beta }}_{02} & \cdots & {\hat{\beta }}_{0p}\\ {\hat{\beta }}_{11} & {\hat{\beta }}_{12} & \cdots & {\hat{\beta }}_{1p}\\ \vdots & \vdots & \vdots \\ {\hat{\beta }}_{F1} & {\hat{\beta }}_{F2} & \cdots & {\hat{\beta }}_{Fp} \end{array}).$$

Equivalently, we may write (Equation 1) as


$$\hat{B}=(\begin{array}{c} {\hat{B}}_{0}\\ {\hat{B}}_{1}\\ \vdots \\ {\hat{B}}_{F} \end{array}),$$

where the *f*-th row vector ${\hat{B}}_{f}$ contains the estimated parameters for **X** *_f_* in the design matrix **X** = (*X*_0_,**X**_1_ *,…*,**X** *_F_*) across all *p* responses. The working responses can then be written in matrix form as


(2) $$\hat{Z}=X\hat{B}+{\hat{R}}^{w},$$

where $\hat{Z}=({\hat{z}}_{1},\ldots ,{\hat{z}}_{p})$ is the matrix of working responses, and ${\hat{R}}^{w}=({\hat{r}}_{1}^{w},\ldots ,{\hat{r}}_{p}^{w})$ contains the corresponding working residuals as column vectors.

By decomposing the design matrix according to the main and interaction effects: **X** = (*X*_0_,**X**_1_ *,…*,**X** *_F_*), the working response matrix in Equation 2 can be rewritten as


(3) $$\begin{array}{l} \hat{Z}=X_{0}{\hat{B}}_{0}+X_{1}{\hat{B}}_{1}+\cdots +X_{F}{\hat{B}}_{F}+{\hat{R}}^{w}\\ \;={\text{M}}_{0}+{\text{M}}_{1}+\cdots +{\text{M}}_{F}+{\hat{R}}^{w}, \end{array}$$

where ${\text{M}}_{f}=X_{f}{\hat{B}}_{f},f=0,1,\ldots ,F$ , are the effect matrices for the different main and interaction terms. To apply the standard ASCA framework, Section (2.2) demonstrates, under certain conditions, the orthogonal decomposition of the sum of squares of the working response matrix into the sum of squares of effect matrices and the sum of squares of working residuals matrix as follows using Equation 3:


(4) $$∥\hat{Z}{∥}^{2}=∥{\text{M}}_{0}{∥}^{2}+∥{\text{M}}_{1}{∥}^{2}+∥{\text{M}}_{2}{∥}^{2}+\cdots +∥{\text{M}}_{F}{∥}^{2}+∥{\hat{R}}^{w}{∥}^{2},$$

where ∥·∥ is the Frobenius norm of a matrix. In the GLM-ASCA decomposition similar to ASCA, Principal Component Analysis is subsequently applied to the various effect matrices. For the *f*-th effect matrix, we obtain


(5) $${\text{M}}_{f}=T_{f}P_{f}^{T},$$

where **T** *_f_* are the scores and **P** *_f_* are the loadings for the effect matrix M**_f_**. Note that when the first few principal components are considered sufficient, a residual term is needed to be added on the right-hand side of Equation 5.

Then, the GLM-ASCA decomposition of all effect matrices is given by


$$\hat{Z}={\text{M}}_{0}+T_{1}P_{1}^{T}+\cdots +T_{F}P_{F}^{T}+{\hat{R}}^{w}.$$

As a result, each main and interaction effect is evaluated using score and loading plots. For example, a plot of the first principal component loadings of effect *f*, **P** *_f_*, shows which responses are most affected by the model effect *f*. Similarly, the score plots of **T** *_f_*, show how the effect levels are located with respect to one another. In addition, projecting the augmented effect matrix helps to show the variability between the observations in the projected score plot (Zwanenburg et al., 2011). Given the augmented matrix,


$${\text{M}}_{\text{f}}^{a}={\text{M}}_{f}+{\hat{R}}^{w},$$

which is analogous to partial residuals in the univariate GLM used for effect display (see **Supplementary Table S1**, **Supplementary Material**), the projected scores are then calculated as


(6) $$T_{f}^{a}={\text{M}}_{f}^{a}P_{f}^{T},\;f=1,2,\ldots ,F.$$

#### 2.1.2 Percentages of variation

For the multivariate case, the ASCA literature offers a modified version of the classical ANOVA approach to calculate the percentage of variance explained by each effect. In ASCA, the square of the Frobenius norm is used to compute the sums of squares of the effect matrices (Vis et al., 2007). In the GLM-ASCA approach, for balanced experimental designs that provide an orthogonal decomposition, as mentioned above (Equation 4), the sum of squares of the working response matrix can be decomposed into the sum of squares of the effect matrices and the sum of squares of the working residual matrix as follows:


$$∥\hat{Z}{∥}^{2}=∥{\text{M}}_{0}{∥}^{2}+∥{\text{M}}_{1}{∥}^{2}+∥{\text{M}}_{2}{∥}^{2}+\cdots +∥{\text{M}}_{F}{∥}^{2}+∥{\hat{R}}^{w}{∥}^{2}.$$

These sums of squares expressed in Frobenius norms can be used to quantify the importance of effects.

That is, the importance of a given effect *f* is determined by the percentage of the total working response variance explained by the effect *f*:


$$\%Var_{f}=\frac{∥{\text{M}}_{f}{∥}^{2}}{∥\hat{Z}{∥}^{2}-∥{\text{M}}_{0}{∥}^{2}}\times 100,\;f=1,2,\ldots ,F,$$

where %*V ar_f_* denotes the percentage of variance explained by effect *f* or importance of effect *f*.

#### 2.1.3 Permutation-based global effect tests

We also explored testing the statistical significance of main and interaction effects across the response variables to support the quantified effect importance. It is possible to determine whether main and interaction effects have a globally significant influence on all response variables by obtaining p-values using permutation testing (Zwanenburg et al., 2011). The advantage of permutation-based tests is that they provide a robust, non-parametric alternative to traditional parametric methods, avoiding reliance on assumptions such as normality, which are often violated in high-dimensional microbiome data. However, this advantage comes at the cost of increased computational burden, particularly when a large number of permutations are required for accurate inference across many features. In the permutation-based global test, we first generate *N_p_* random permutations (*Y* ^(^ *^k^* ^)^, *k* = 1,2*,…,N_p_*) of the rows of the response data matrix *Y*. For each permuted dataset, a GLM is fitted, the effect matrices are retrieved, PCA is applied to the effect matrices, and the score and loading matrices are obtained for both the observed data *Y* and all permuted datasets *Y* ^(^ *^k^* ^)^. The first *q* principal components can be used for permutation testing, visualization, and further analysis. The number of principal components (*q*) retained plays a critical role in test sensitivity and is generally chosen based on criteria such as cumulative explained variance (e.g., ≥80%) or through visual inspection of scree plots. Formal statistical tests may also be used to determine *q* (Camargo, 2022, and references therein). Importantly, *q* does not need to be the same for all effect matrices, as each effect may explain a different proportion of total variance and require separate consideration.

A global statistic is defined based on the square Frobenius norm of the score matrix of the first *q* principal components, ${\text{T}}_{{\text{f}}_{q}}$ . For testing effect *f*, the statistic for the observed data is then computed as


$$SS_{f}=∥{\text{T}}_{{\text{f}}_{q}}{∥}^{2},$$

and for all permuted datasets under the null distribution as


$$SS_{f}^{\left(k\right)}=∥{\text{T}}_{{\text{f}}_{q}}^{\left(k\right)}{∥}^{2},\;k=1,\ldots ,N_{p}.$$

Finally, the p-value for effect of *f* is calculated as


$$\text{p}-{\text{value}}_{f}=\frac{\#\{SS_{f}^{\left(k\right)}\geq SS_{f},\;\;\;\;\;k=1,\ldots ,N_{p}\}+1}{N_{p}+1},\;f=1,\cdots ,F,$$

and the null hypothesis of no effect is rejected if the resulting p-value is less than a specified significance level (e.g., 0.05).

#### 2.1.4 Feature selection in GLM-ASCA

When an effect is found to be significant using the permutation-based global effect test, the next step is to identify features that contribute to the significant effect. In classical PCA, a rule of thumb can be used to select features with high absolute loadings satisfying a specified threshold criterion. Alternatively, sparse PCA can be used to set the loadings of unimportant features to zero (Saccenti et al., 2018). In the ASCA context, for example, group-wise ASCA (GASCA) incorporates sparsity based on group-wise principal component analysis, where sparsity is defined in terms of groups of correlated variables identified in the correlation matrices computed from the effect matrices (Saccenti et al., 2018). In addition, several permutation-based significance tests have been implemented in the ASCA framework. For example, tests based on leverages and squared prediction errors are discussed in ASCA-genes (Tarazona et al., 2012; Nueda et al., 2007).

In this work, we used scaled leverages that measure the importance of features in a PCA model, computed as


(7) $$h_{f}=\text{diag}(C_{f}C_{f}^{T}),\;f=1,2,\ldots ,F,$$

where **h** *_f_* is a vector of scaled leverages corresponding to each feature in the PCA model for effect *f*, diag denotes the diagonal entries of a matrix, and **C** *_f_* is a matrix of scaled loadings obtained by multiplying the loadings **P** *_f_* of the first *q* principal components by the square roots of the variances explained by the respective principal components. When there are two or more principal components, this adjustment to the unscaled leverages $\left(\text{diag}(P_{f}P_{f}^{T})\right)$ (Tarazona et al., 2012; Nueda et al., 2007), takes into account the variance contribution of each principal component to feature selection.

Based on the permutation procedure described above for global effect tests and the computed PCA loadings, we assess the importance of each feature for a given effect. To test the contribution of feature *j* to effect *f*, we compute the scaled leverage statistic from the observed data, given by the *j*-th element of **h** *_f_* in Equation 7 and denoted by **h** *_fj_*.

Under the null distribution, for each permuted dataset *k* = 1*,…,N_p_*, we compute the permuted scaled leverage statistics:


$$h_{f}^{\left(k\right)}=\text{diag}(C_{f}^{\left(k\right)}C_{f}^{(k{)}^{T}}),\;f=1,2,\ldots ,F,$$

where $C_{f}^{\left(k\right)}$ is the matrix of scaled loadings obtained by multiplying the permutation-based loadings $P_{f}^{\left(k\right)}$ (from the first *q* principal components) by the square roots of the variances they explain. The permuted scaled leverage statistic for feature j is the j -th element, $h_{fj}^{\left(k\right)}$ , of $h_{f}^{\left(k\right)}$ .

The p-value for testing the contribution of feature *j* to effect *f* is then calculated as:


$$\text{p}-{\text{value}}_{fj}=\frac{\#\{h_{fj}^{\left(k\right)}\geq h_{fj}\}+1}{N_{p}+1},\;f=1,\ldots ,F,\text{ and }j=1,\ldots p.$$

Finally, to account for multiple testing of *p* features on effect *f*, we apply the Benjamini-Hochberg (BH) procedure to control the false discovery rate (FDR) (Benjamini and Hochberg, 1995). Features with BH adjusted p-values below a predefined significance level (e.g., 0.05) are identified as significantly contributing to the corresponding effect.

### 2.2 Orthogonal decomposition in GLM

This section presents the technical details of the orthogonal decomposition in GLMs provided in (4). We demonstrate the orthogonal decomposition of the sum of squares of the working response variable by showing that the working residuals are orthogonal to the fitted values in balanced designs where the design matrix **X** is orthogonal with respect to effects. It has been shown that in Generalized Linear Models, the adjusted working residuals $\left(r^{w*}={\hat{W}}^{1/2}{\hat{r}}^{w}\right)$ , like the linear model ordinary residuals, provide an exact orthogonal decomposition of the sum of squares of the adjusted working response $\left({\hat{z}}^{*}={\hat{W}}^{1/2}{\hat{z}}^{w}\right)$ (Lovison, 2014):


(8) $${\hat{z}}^{*T}{\hat{z}}^{*}={\hat{\eta }}^{*T}{\hat{\eta }}^{*}+{\hat{r}}^{w*T}{\hat{r}}^{w*}.$$

The main challenge in extending ASCA models directly to GLMs, similar to their use in LMs, is the difficulty in further orthogonally decomposing the linear predictor $\left({\hat{\eta }}^{*}\right)$ in Equation 8 to specific effect sources of variation. That is, in GLM, unless the observation weights are one or constant, further orthogonal effect decomposition of ${\hat{\eta }}^{*}={\hat{W}}^{1/2}\hat{\eta }={\hat{W}}^{1/2}X\hat{\beta }$ according to orthogonal columns of a design matrix **X** is not straightforward (Hosmer et al., 2013). To address this problem, we considered two orthogonal decomposition issues: decomposing the sum of squares of the working response and decomposing the sum of squares of the linear predictor.

Decomposing the sum of squares of the working response: we establish an exact orthogonal decomposition using the unscaled or working response $\hat{z}$ rather than the scaled or adjusted working response $\left({\hat{z}}^{*}\right)$ . That is, the sum of squares of the working response can be decomposed into the sum of squares of the linear predictor and the sum of squares of the working residuals:


(9) $${\hat{z}}^{T}\hat{z}={\hat{\eta }}^{T}\hat{\eta }+{\hat{r}}^{wT}{\hat{r}}^{w}.$$

This can be demonstrated as follows. Using definitions of the linear predictor we have


(10) $$\begin{array}{l} \hat{\eta }=X\hat{\beta }\\ \;=X{\left(X^{T}\hat{W}X\right)}^{-1}X^{T}\hat{W}\hat{z}\\ \;={\hat{W}}^{-1/2}{\hat{W}}^{1/2}X{\left(X^{T}\hat{W}X\right)}^{-1}X^{T}{\hat{W}}^{1/2}{\hat{W}}^{1/2}\hat{z}\\ \;={\hat{W}}^{-1/2}\hat{H}{\hat{W}}^{1/2}\hat{z},\text{ where the Hat matrix }\hat{H}\\ \;={\hat{W}}^{1/2}X{\left(X^{T}\hat{W}X\right)}^{-1}X^{T}{\hat{W}}^{1/2}\\ \;=G\hat{z}, \end{array}$$

Where


(11) $$G={\hat{W}}^{-1/2}\hat{H}{\hat{W}}^{1/2}.$$

Similarly, for the working residuals


(12) $$\begin{array}{l} {\hat{r}}^{w}=\hat{z}-\hat{\eta }\\ \;=\hat{z}-G\hat{z}\\ \;=(I-G)\hat{z}. \end{array}$$

In general, the sum of squares of the working response decomposes as


$${\hat{z}}^{T}\hat{z}={\hat{\eta }}^{T}\hat{\eta }+{\hat{r}}^{wT}{\hat{r}}^{w}+2{\hat{\eta }}^{T}{\hat{r}}^{w}.$$

So that, orthogonality of $\hat{\eta }$ and ${\hat{r}}^{w}$ can be achieved if ${\hat{\eta }}^{T}{\hat{r}}^{w}=0$ . Using Equations 10, 12 we obtain

${\hat{\eta }}^{T}{\hat{r}}^{w}={\left(G\hat{z}\right)}^{T}(I-G)\hat{z}$


(13) $$={\hat{z}}^{T}(G^{T}-G^{T}G)\hat{z}.$$

We note that the orthogonality property can be satisfied if the matrix **G** is idempotent and symmetric like the Hat matrix, $\hat{H}$ . However, from the properties of $\hat{H}$ , it is clear that $\hat{G}$ is idempotent but not symmetric. That is


$$GG={\hat{W}}^{-1/2}\hat{H}{\hat{W}}^{1/2}{\hat{W}}^{-1/2}\hat{H}{\hat{W}}^{1/2}={\hat{W}}^{-1/2}\hat{H}{\hat{W}}^{1/2}=G.$$

demonstrates that **G** is idempotent but


$$G^{T}={\left({\hat{W}}^{-1/2}\hat{H}{\hat{W}}^{1/2}\right)}^{T}={\hat{W}}^{1/2}\hat{H}{\hat{W}}^{-1/2},$$

differs from **G** implying that **G** is not symmetric under the given general setting of GLMs with observation weights $\hat{W}$ , posing a challenge to achieving orthogonality. However, here two approaches are introduced to ensure orthogonality in GLMs. The first approach utilizes the properties of the Hat matrix derived from balanced treatment designs and saturated model formulations. The second approach involves choosing or deriving a link function in GLMs that provides observation weights equal to one or a constant value.

#### 2.2.1 Balanced designs and saturated models

First, we consider the properties of the Hat matrix to establish an orthogonal decomposition of the sum of squares of the working response as the sum of squares of the working residuals and the fitted linear predictor values. As shown in Equation 11, given a special structure of the Hat matrix $\hat{H}$ and weight matrix $\hat{W}$ that allow these matrices to be commutative under multiplication, then **G** = $\hat{H}$ can be established.

With some algebraic simplifications using the full rank property of the design matrix arising from balanced designs and saturated models for one replication per experimental unit (see examples in the **Supplementary Material Text S2**), the Hat matrix turns out to be the identity matrix of dimension *n* × *n*. That is, using the design matrix **X** ^(1)^ for one replication per experimental unit:


(14) $$\begin{array}{l} \hat{H}={\hat{W}}_{n}^{1/2}X^{\left(1\right)}{\left(X^{(1)T}{\hat{W}}_{n}X^{\left(1\right)}\right)}^{-1}X^{(1)T}{\hat{W}}_{n}^{1/2}\\ \;={\hat{W}}_{n}^{1/2}X^{\left(1\right)}{\left(X^{(1)T}{\hat{W}}_{n}X^{\left(1\right)}\right)}^{-1}X^{(1)T}{\hat{W}}_{n}^{1/2}\;\times ({\hat{W}}_{n}^{1/2}X^{\left(1\right)}){\left({\hat{W}}_{n}^{1/2}X^{\left(1\right)}\right)}^{-1}\\ \;={\hat{W}}_{n}^{1/2}X^{\left(1\right)}{\left(X^{(1)T}{\hat{W}}_{n}X^{\left(1\right)}\right)}^{-1}(X^{(1)T}{\hat{W}}_{n}X^{\left(1\right)}){\left({\hat{W}}_{n}^{1/2}X^{\left(1\right)}\right)}^{-1}\\ \;={\hat{W}}_{n}^{1/2}X^{\left(1\right)}I_{n\times n}{\left({\hat{W}}_{n}^{1/2}X^{\left(1\right)}\right)}^{-1}\\ \;=I_{n\times n}. \end{array}$$

In this case, it follows that the matrix **G** also equals the identity matrix,


$$G={\hat{W}}^{-1/2}\hat{H}{\hat{W}}^{1/2}={\hat{W}}^{-1/2}I_{n\times n}{\hat{W}}^{1/2}={\hat{W}}^{-1/2}{\hat{W}}^{1/2}=I_{n\times n}.$$

In general, for *R* replications included in the experiment, the design matrix data structure can be expressed by vertically concatenating the **X** ^(1)^ coding matrices as


$$\text{X}=\begin{array}{c} B_{1}\\ B_{2}\\ \vdots \\ B_{R} \end{array}(\begin{array}{c} {\text{X}}^{\left(1\right)}\\ {\text{X}}^{\left(1\right)}\\ \vdots \\ {\text{X}}^{\left(1\right)} \end{array}),$$

where *B_i_* is used to indicate a block of *n* experimental units for the *i*-th replication. Similarly, the weights can be expressed as a block diagonal matrix as


$$\hat{\text{W}}=\begin{array}{c} B_{1}\\ B_{2}\\ \vdots \\ B_{R} \end{array}(\begin{array}{ccccc} {\hat{\text{W}}}_{n} & 0 & 0 & \cdots & 0\\ 0 & {\hat{\text{W}}}_{n} & 0 & \cdots & 0\\ \vdots & \vdots \\ 0 & 0 & 0 & \cdots & {\hat{\text{W}}}_{n} \end{array}).$$

Letting


$$Q={\hat{W}}^{1/2}X={\left({\hat{W}}_{n}^{1/2}X^{\left(1\right)},{\hat{W}}_{n}^{1/2}X^{\left(1\right)},\cdots ,{\hat{W}}_{n}^{1/2}X^{\left(1\right)}\right)}^{T},$$

the Hat matrix is rewritten as


$$\hat{H}=Q{\left(Q^{T}Q\right)}^{-1}Q^{T},$$

and using Equation 14 obtained for one replication per experimental unit, we have the final structure of the Hat matrix which is a matrix of identical identity block matrices multiplied by the fraction of replications $\frac{1}{R}$ (detail derivation is given in the **Supplementary Information, Text S3**)


$$\hat{H}=(\begin{array}{ccc} \frac{1}{R}I_{n\times n} & \cdots & \frac{1}{R}I_{n\times n}\\ \vdots & \vdots \\ \frac{1}{R}I_{n\times n} & \cdots & \frac{1}{R}I_{n\times n} \end{array}).$$

Similar Hat matrix structures are also described for ANOVA fixed effect models (Orenti et al., 2012). Examples of Hat matrices in balanced and saturated designs in GLMs are given in **Text S4** (**Supplementary Material**).

We now finalize the orthogonality property in GLMs. For balanced designs and saturated models, we carefully exploit the block identity structure of the Hat matrix derived above and the diagonal form of the weight matrix to do commutative multiplication of the matrices in **G**:


$$\begin{array}{l} G={\hat{W}}^{-1/2}\hat{H}{\hat{W}}^{1/2}\\ \;\;\;\;\;=\hat{H}{\hat{W}}^{-1/2}{\hat{W}}^{1/2},\text{ since }\hat{H}\text{ includes block identity or block diagonal matrices of ones and }\hat{W}\text{ is a diagonal block matrix}\\ \;=\hat{H}. \end{array}$$

It follows that, for saturated models and balanced designs, **G** is idempotent and symmetric. Next, we simplify the orthogonal condition in Equation 13


$$\begin{array}{l} {\hat{\eta }}^{T}{\hat{r}}^{w}={\hat{z}}^{T}G^{T}(I-G)\hat{z}\\ ={\hat{z}}^{T}(G-G)\hat{z},\;G\;\text{is}\;\text{symmetric}\;\text{and}\;\text{idempotent}=0. \end{array}$$

Thus, $\hat{\eta }$ and $r^{w}$ are orthogonal. As a result, we obtain an exact orthogonal decomposition of the sum of squares of the working response:


$${\hat{z}}^{T}\hat{z}={\hat{\eta }}^{T}\hat{\eta }+{\hat{r}}^{wT}{\hat{r}}^{w}.$$

Introducing new link functions with weights equal to one or a constant

The second approach to ensure orthogonality is to set the observation weights to 1 or a constant value (Dossou-Gbete and Tinsson, 2005). Under this constraint, $G=\hat{H}$ and orthogonality follows as described above, or the scaled version of the orthogonal decomposition in Equation 8 simplifies to the unscaled orthogonal decomposition in Equation 9. The constraint can be met by introducing a new link function with observation weights *w_i_* = *w, i* = 1*,…, n* where *w* = 1 or a constant. Using the definition of observation weights which can be expressed as


(15) $$w=\frac{1}{{\left(\frac{\partial g(\mu )}{\partial \mu }\right)}^{2}V(\mu )},$$

With weights equal to a constant, a new link function can be derived (Dossou-Gbété and Tinsson, 2005) by simplifying Equation 15 as


$$g(\mu )=\frac{1}{w^{1/2}}\int V{\left(\mu \right)}^{-1/2}\;d\mu .$$

In general, we rewrite the resulting orthogonal decomposition of the squared norm of $\hat{z}$ as


(16) $$∥\hat{z}{∥}^{2}=∥\hat{\eta }{∥}^{2}+∥{\hat{r}}^{w}{∥}^{2}.$$

Decomposing the sum of squares of the linear predictor $\left(\hat{\eta }\right)$ : we now address the second issue of orthogonality, which is an orthogonal effect decomposition of the linear predictor $\hat{\eta }$ into specific effect sources of variation. For a balanced design with a model containing all *F* main and interaction effects, and using sum coding of factor levels, the design matrix **X** can be decomposed into *F* + 1 orthogonal blocks that include the constant term and one for each model effect: **X** = (*X*_0_ | **X**_1_ |*…* | **X** *_F_*). The design matrix **X** being orthogonal leads to further orthogonal decomposition of $\hat{\eta }=X_{0}{\hat{\beta }}_{0}+X_{1}{\hat{\beta }}_{1}+\cdots X_{F}{\hat{\beta }}_{F}$ as


(17) $$∥\hat{\eta }{∥}^{2}=∥X_{0}{\hat{\beta }}_{0}{∥}^{2}+∥X_{1}{\hat{\beta }}_{1}{∥}^{2}+∥X_{2}{\hat{\beta }}_{2}{∥}^{2}+\cdots +∥X_{F}{\hat{\beta }}_{F}{∥}^{2}.$$

When the link function corresponds to observation weights equal to one or a constant, a balanced design alone is a sufficient condition to ensure this orthogonal decomposition, consistent with the classical regression partitioning of the sum of squares (Montgomery et al., 2021; Radhakrishna Rao and Toutenburg, 1999). For completeness of presentation, details of the derivations under our framework are presented in Text S5 (**Supplementary Material**). Consequently, in the context of GLM, a full orthogonal decomposition of the sum of squares of the working response is obtained by substituting Equation 17 into Equation 16 as


$$∥\hat{z}{∥}^{2}=∥X_{0}{\hat{\beta }}_{0}{∥}^{2}+∥X_{1}{\hat{\beta }}_{1}{∥}^{2}+∥X_{2}{\hat{\beta }}_{2}{∥}^{2}+\cdots +∥X_{F}{\hat{\beta }}_{F}{∥}^{2}+∥{\hat{r}}^{w}{∥}^{2}.$$

For example, for a two-factor model with main effects A and B and interaction effect AB the GLM decomposition is


$$∥\hat{z}{∥}^{2}=∥X_{0}{\hat{\beta }}_{0}{∥}^{2}+∥X_{A}{\hat{\beta }}_{A}{∥}^{2}+∥X_{B}{\hat{\beta }}_{B}{∥}^{2}+∥X_{AB}{\hat{\beta }}_{AB}{∥}^{2}+∥{\hat{r}}^{w}{∥}^{2}.$$

In this study, we work under the assumption of a balanced design and a saturated model, which enables orthogonal decomposition and facilitates interpretable effect estimation within the GLM-ASCA framework described in Section 2.1.1. Although this assumption is strong, it is frequently satisfied in well-controlled experimental settings, particularly in randomized factorial designs, where balance is intentionally maintained to ensure equal representation of treatment combinations, minimize confounding, and support orthogonal design structures that allow precise and independent estimation of factor effects. We also observed that incorporating an offset term in the GLM violates the orthogonal decomposition property. As part of our ongoing research, we are developing extensions to the GLM-ASCA framework to accommodate unbalanced designs and non-orthogonal decompositions, with the goal of increasing its applicability to more diverse and complex experimental settings.

### 2.3 GLM for microbiome data analysis

Analyzing microbiome datasets from high-throughput sequencing presents challenges due to overdispersion, zero inflation, non-normality, and compositionality. We addressed these issues using generalized linear models (GLMs) with a Tweedie family of distributions—one of the more flexible and general families for count and positive continuous data analysis. Compared with negative binomial–based models (Love et al., 2014b; Robinson et al., 2010), which are sensitive to zero inflation, and zero-inflated models (Zhang et al., 2016b), which require separate components for modeling excess zeros and abundance, the Tweedie GLM offers an integrated approach that inherently accommodates both zero and nonzero values within a single framework. This eliminates the need for an additional zero inflation term or arbitrary data imputations. Furthermore, the Tweedie model avoids the necessity of adding pseudocounts (Xia, 2023), which is typically required when applying log transformation in Gaussian-based models.

The Tweedie distribution is parameterized by the mean (*µ*), dispersion (*ϕ*), and power parameter (*ρ*), and it yields several well-known distributions for specific values of ρ, such as Gaussian (*ρ* = 0), Poisson (*ρ* = 1), gamma (*ρ* = 2), inverse Gaussian (*ρ* = 3), and compound Poisson–gamma (1 *< ρ <* 2). In particular, the Tweedie compound Poisson–gamma distribution has a point mass at zero and a skewed distribution on the positive real line, making it suitable for modeling count and positive continuous data with excess zeros, such as microbiome sequence count data (Mallick et al., 2022). This distribution has been applied for differential expression analysis of single-cell RNA sequencing (scRNA-seq) data (Mallick et al., 2022). The Tweedie compound Poisson–gamma distribution (Dunn and Smyth, 2005; Lian et al., 2023) is given by


$$f(y;\mu ,\phi ,\rho )=a(y;\phi ,\rho )\text{ exp }\{\frac{1}{\phi }(\frac{y\mu^{1-\rho }}{1-\rho }-\frac{\mu^{2-\rho }}{2-\rho })\},$$

where the form of *a*(*y,ϕ,ρ*) is found in (Dunn and Smyth, 2005).

We considered GLM with a Tweedie distribution for each response, *Y* ∼ Tweedie(*µ,ϕ,ρ*), with logarithm link function *g*(*µ*) = log(*µ*) = *η* = **x** *^T^β*, then for given *ϕ* and *ρ*, the mean and variance to be used in the GLM setting are given by Equation 18

$E(Y)=\mu =g^{-1}(\eta )$


(18) $$Var(Y)=\phi \mu^{\rho }.$$

Furthermore, the compositional nature of microbiome data—resulting from differences in total sequence read counts (sequencing depth or library size) across samples due to the sequencing process—is addressed through normalization methods. Normalization is an important step in microbiome sequencing data analysis used to remove bias caused by compositional effects or differences in sequencing depths or library sizes between samples. Several forms of normalization have been introduced (Xia, 2023) for microbiome data, including scaling-based normalization, zero-inflation–based normalization, and compositionally aware normalization. Some commonly used scaling-based normalization procedures, originally adopted from RNA sequencing (RNA-seq) data analysis, include the median-of-ratios method (Love et al., 2014b) and the trimmed mean of M-values (TMM) (Robinson et al., 2010). In addition, methods such as the geometric mean of pairwise ratios (Chen et al., 2018), Wrench normalization (Kumar et al., 2018), and the geometric mean of positive counts (poscounts) (McMurdie and Holmes, 2013) have been extended to account for zero inflation. Scaling-based normalization involves obtaining a scaling factor that adjusts raw counts to produce normalized counts or normalized library sizes. Normalized library sizes, for example, are used as offsets in generalized linear models (GLMs) to remove biases caused by differences in sequencing depths (Love et al., 2014b; Robinson et al., 2010; Mallick et al., 2022). On the other hand, compositionally aware normalization methods commonly used include centered log-ratio (CLR) transformation (Fernandes et al., 2014) and additive log-ratio (ALR) transformation (Mandal et al., 2015).

In this work, the raw microbiome count data were normalized using either the “poscounts” option in the *DESeq2* R package (Love et al., 2014a) or transformed using the modified centered log-ratio transformation (mCLR) from the *SPRING* R package (Yoon et al., 2019). By using normalized or transformed counts that account for biases caused by compositional effects or variations in library size, including an offset term is not necessary in the GLM. This approach maintains the orthogonal decomposition outlined above within the Tweedie GLM framework.

In general, the Tweedie GLM, which is appropriate for modeling positive data with many zeros, can effectively address the key characteristics of microbiome data. The Tweedie compound Poisson–gamma model (1 *< ρ <* 2), implemented in the R packages tweedieverse (Mallick et al., 2022) for the analysis of overdispersed and zero-inflated single-cell RNA-seq count data and mcglm (Bonat and Jørgensen, 2016), was used to estimate the model parameters.

### 2.4 Plant microbiome data: experimental setup and microbial DNA extraction

To assess the impact of nitrogen availability on the bacterial community composition of tomato roots, tomato seeds (Solanum lycopersicum cv. Moneymaker) were grown in an aeroponic system following the methodology outlined by Abedini et al. (manuscript in preparation^1^). Briefly, the seeds underwent surface sterilization and were pre-germinated at 25°C for 3 days. The pre-germinated seeds were then transplanted into small baskets filled with greenhouse soil. These baskets were placed in a large bucket equipped with an aeroponic system (**Supplementary Figure S1**). The aeroponic system utilized one-quarter–strength Hoagland solution, with a spraying duration of 15 s and a 10 min interval between sprays. The greenhouse environment was maintained at 22°C with 60% relative humidity and an 8 h dark/16 h light photoperiod. After 10 days of growth under standard control conditions, nitrogen starvation was initiated. The plants were randomly assigned to two groups: the control group, which received one-quarter–strength Hoagland solution containing 5.6 mM nitrogen, and the nitrogen-starved group, which received one-quarter–strength Hoagland solution without NH_4_NO_3_. Sampling occurred at 4, 8, 12, and 16 days after the start of nitrogen starvation, with five replicates for both the control and nitrogen-starved conditions. Because control and nitrogen-starved plants were grown and harvested at the same time for each sampling point, observed differences in the root microbiome between the two conditions can be attributed to nitrogen deprivation rather than to variations in growth stage.

Afterward, 45 mL of the collected Hoagland solution was vacuum filtered through a 0.2 *µ*m membrane filter. The resulting filter, which retained the microbes, was placed into a PowerSoil kit bead tube and processed using a bead mill Tissuelyser for 5 min. Microbial DNA was then extracted following the PowerSoil protocol. The extracted genomic DNA was amplified for bacterial 16S rDNA targeting the V3 and V4 regions using primers 341F (5′-CCTACGGGNGGCWGCAG-3′) and 805R (5′-GACTACHVGGGTATCTAATCC-3′). Sequencing was performed on the Illumina NovaSeq6000 SP platform at Genome Quebec in Montreal, Quebec. Sample demultiplexing was carried out at the Genome Quebec facility. The resulting sequences underwent trimming, quality assessment, merging, and taxonomic classification using the dadasnake pipeline (Weißbecker et al., 2020). Taxonomic classification was performed with mothur using SILVA SSU v138 as the reference database. After preprocessing, the 16S dataset retained a total of 5300 amplicon sequence variants (ASVs) across five replicates for each of the four time points (4, 8, 12, and 16 days) under both control and nitrogen-starved conditions, resulting in a total of 40 samples.

### 2.5 Simulation study

We conducted a simulation study to evaluate the effectiveness of GLM-ASCA in identifying true positive taxa associated with main and interaction effects. To achieve this, we generated synthetic microbiome data based on experimental conditions consisting of four time points, two treatment conditions, and eight levels for the time-treatment interaction.

Our simulation approach used the R package SparseDOSSA2 (Ma et al., 2021), which operates independently of the distributional assumptions underlying the Tweedie GLM. SparseDOSSA2 is a statistical simulation framework that can be adapted to analyze plant microbiome datasets, effectively capturing plant microbial dynamics, as demonstrated previously in human microbiome studies (Ma et al., 2021). SparseDOSSA2 generates realistic simulated data by parameterizing real-world template microbial datasets to reflect key microbiome characteristics such as zero inflation and overdispersion. For our study, we used the experimental plant microbiome data described in Section 2.4 as a template. First, SparseDOSSA2 was used to estimate taxon-specific parameters with a Bayesian hierarchical model, including the means and variances of nonzero log abundances, as well as the probabilities of zeros ( $\tilde{\text{µ}}$ *_j_,σ_j_* ^2^ *,π_j_,j* = 1*,…,p*). These estimated parameters were then used to generate synthetic features from a zero-inflated, truncated log-normal distribution. Accordingly, we produced null (baseline) data consisting of 206 features and 20 samples under control (nitrogen-rich) conditions. Strict filtering criteria were applied to retain features with at least 10 counts in a minimum of five samples.

Next, to simulate taxa with differential effects, we incorporated the plant microbiome experimental conditions, which included the main effects growth condition (control and N-starvation) and time (4, 8, 12, and 16 days), as well as the interaction effect between growth condition and time. We then selected 45 of the 206 null taxa with a relative abundance of at least 20% to be spiked-in as having “true” differential main and interaction effects with known effect sizes (log-fold differences). Of the 45 taxa with “true” differential effects, 20 were assigned growth condition main effects (*β*_1_), 15 were assigned time effects represented by three parameters (*β*_2_, *β*_3_, *β*_4_), and 10 were assigned growth condition × time interaction effects represented by three parameters (*β*_5_, *β*_6_, *β*_7_). The effect size parameter values were varied: half of the spiked features were assigned positive effect sizes (*β*_1_ *,β*_2_ *,β*_3_ *,β*_4_ *,β*_5_ *,β*_6_ *,β*_7_) = (2,3,1.5,1.5,2.8,2.4,1.4), and the other half were assigned negative effect sizes (−2,−3,−1.5,−1.5,−2.8,−2.4,−1.4). To account for these effects on the data generation by SparseDOSSA2, the taxon-specific mean log abundances ( $\tilde{\mu }$ *_j_*) used to generate null features were modified to mean log abundances $\tilde{\mu }$ *_ij_* across samples for the spiked-in features using


$$\underset{\begin{array}{l} \text{Mean log abundance}\\ \text{ across samples} \end{array}}{\underset{︸}{{\tilde{\text{μ}}}_{\text{ij}}}}=\underset{\begin{array}{l} \text{ Taxon specific}\\ \text{mean log abundance} \end{array}}{\underset{︸}{{\tilde{\text{μ}}}_{\text{j}}}}+\underset{\text{Growth conditon effect}}{\underset{︸}{{\text{β}}_{1}{\text{x}}_{1\text{i}}}}+\underset{\text{Time effect}}{\underset{︸}{{\text{β}}_{2}{\text{x}}_{2\text{i}}+{\text{β}}_{3}{\text{x}}_{3\text{i}}+{\text{β}}_{4}{\text{x}}_{4\text{i}}}}+\underset{\text{Interaction effect}}{\underset{︸}{{\text{β}}_{5}{\text{x}}_{5\text{i}}+{\text{β}}_{6}{\text{x}}_{6\text{i}}+{\text{β}}_{7}{\text{x}}_{7\text{i}}}}$$

where *x* represents values assigned through sum or deviation coding for the factors: growth condition, time, and their interaction. The spiked-in taxa are then generated based on zero-inflated truncated log-normal distribution with ( $\tilde{\mu }$ *_ij_,σ_j_* ^2^ *,π_j_,i* = 1*,…,n*; *j* = 1*,…,p*).

The sample sizes were varied at 40, 80, and 160, with 5, 10, and 20 replications, respectively. We generated 100 simulated abundance datasets, each containing 45 spiked-in taxa (“true positives”) and 161 null taxa with no differential effect (“true negatives”). Of the 45 spiked taxa, 20, 15, and 10 were true positives for growth condition, time, and growth condition × time interaction effects, respectively. The efficacy of Tweedie GLM-ASCA in identifying taxa with differential effects in high-dimensional microbiome data was evaluated using these synthetic datasets with known (“true”) effect sizes. We compared GLM-ASCA with two recently developed methods for microbiome data analysis, MaAsLin2 (Mallick et al., 2021) and LinDA (Zhou et al., 2022). Both accommodate multiple continuous and categorical covariates, unlike many differential abundance methods that consider only a single categorical covariate. MaAsLin2 utilizes generalized linear models to identify multivariable associations between microbial features and metadata, whereas LinDA applies linear models for differential abundance analysis, accounting for compositional bias and inflated zeros in microbiome data.

## 3 Results

### 3.1 Simulation results

To evaluate the performance of GLM-ASCA in microbiome data analysis, we conducted a simulation study generating 100 simulated microbiome datasets including 206 taxa with varying total sample sizes (40, 80, and 160), as described in Section 2.5. After fitting GLM-ASCA, BH-adjusted p-values were used to classify taxa as true positives (TP), false positives (FP), true negatives (TN), and false negatives (FN). Performance was evaluated based on sensitivity (statistical power), specificity, FDR, area under the curve (AUC), F1-score (F-score), and Matthews correlation coefficient (MCC).

#### 3.1.1 Performance of GLM-ASCA with different normalization methods

**Figure 1:**
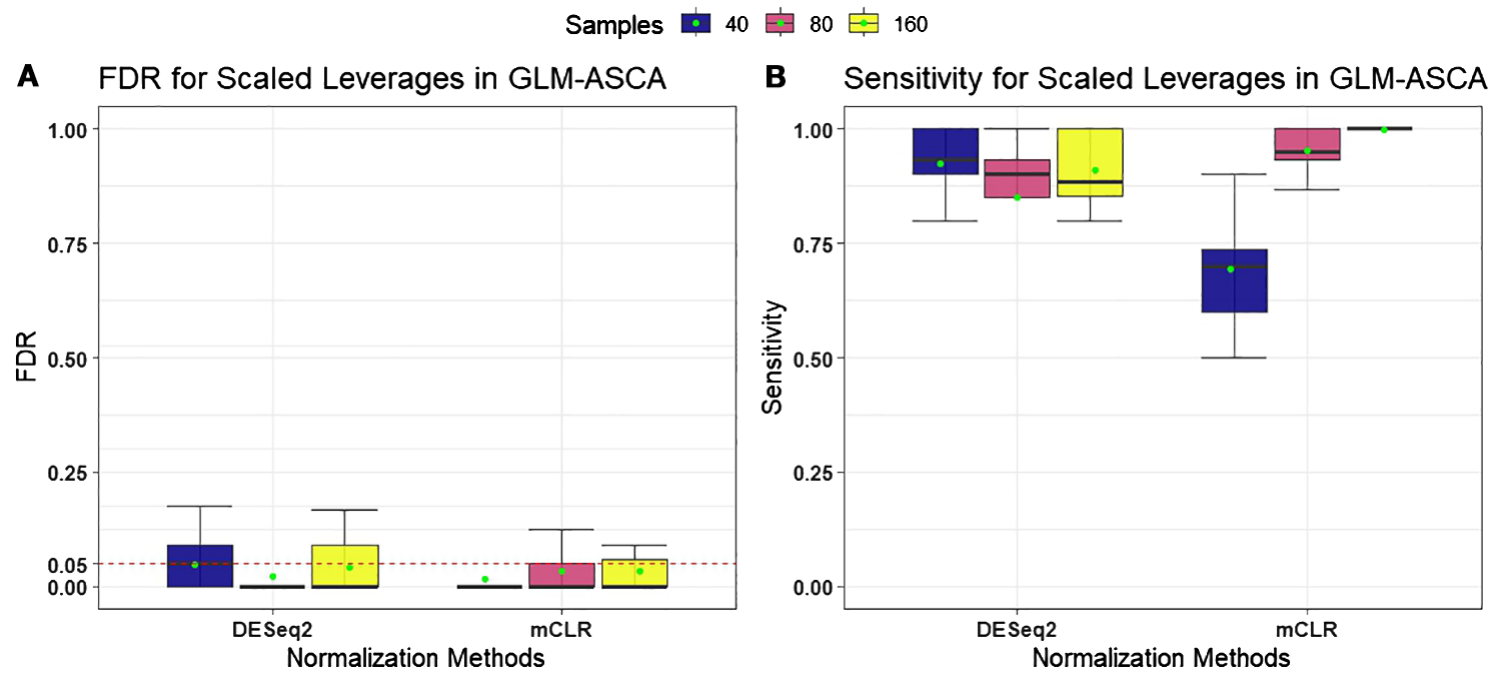
Performance measures [false discovery rate (FDR) and statistical power (sensitivity)] for GLM–ASCA using DESeq2 (poscounts) and mCLR normalizations, evaluated on simulated data derived from a template plant microbiome dataset. Boxplots are colored by total sample size. **(A)** With both normalizations, GLM-ASCA with scaled-leverage–based permutation feature selection demonstrated mean FDR (green dots) close to the nominal 5% level. **(B)** GLM-ASCA with DESeq2 (poscounts) achieved higher power in small-sample scenarios, whereas GLM-ASCA with mCLR achieved higher power in large-sample scenarios.

We first evaluated the performance of GLM-ASCA using two normalization methods: “poscounts” from *DESeq2* and mCLR from *SPRING*, incorporating permutation-based feature selection with scaled leverages. Performance was assessed based on FDR control and statistical power (sensitivity) to detect relationships between spiked taxa and the effects of growth condition, time, and their interaction. As shown in **Figure 1**, GLM-ASCA with DESeq2-based normalization and scaled-leverage feature selection exhibited superior statistical power compared with mCLR normalization, particularly in scenarios with small sample sizes. However, both normalization methods effectively controlled FDR at the nominal 5% level across all sample size settings. Detailed performance measures of GLM-ASCA for individual main and interaction effects are presented in **Supplementary Figure S2**.

#### 3.1.2 Comparison of GLM-ASCA with alternative methods

To further assess GLM-ASCA, we compared its performance with MaAsLin2 and LinDA. In these comparisons, GLM-ASCA was implemented using “poscounts” normalization, while MaAsLin2 was applied with its default settings except for normalization, which was adjusted to CSS (cumulative sum scaling) to address zero inflation in microbiome data. LinDA was used with all default settings.

**Figure 2:**
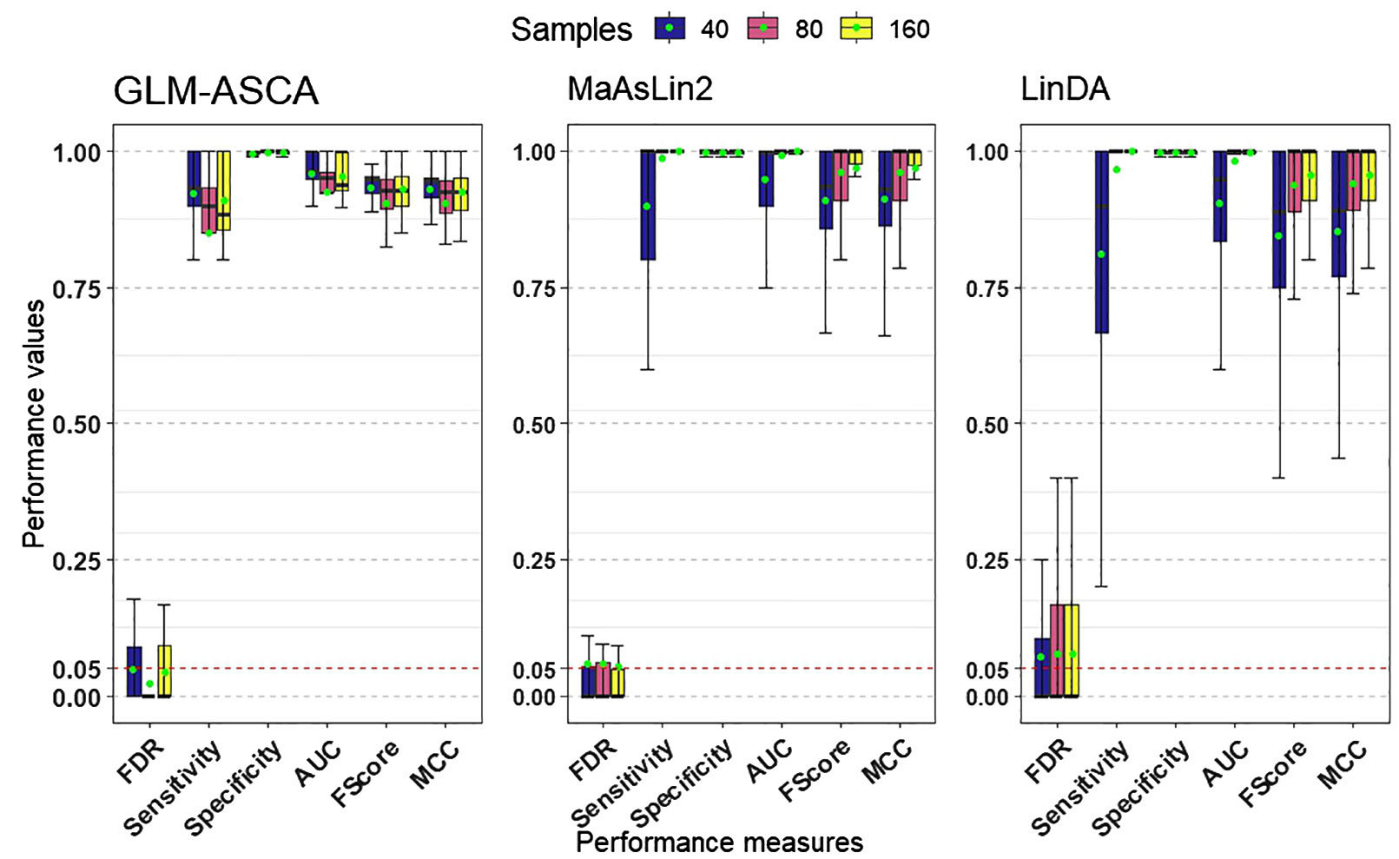
Performance measures (FDR, statistical power [sensitivity], specificity, AUC, F1-score, and MCC) for GLM–ASCA, MaAsLin2, and LinDA, evaluated on simulated data derived from a template plant microbiome dataset. Boxplots are colored by total sample size. GLM-ASCA, MaAsLin2, and LinDA demonstrated mean FDR (green dots) close to the nominal 5% level while maintaining high statistical power, AUC, F1-score, and MCC. GLM–ASCA achieved higher power in small-sample scenarios.

**Figure 2** presents the simulation results across multiple performance measures. In our simulations, all three methods—GLM-ASCA, MaAsLin2, and LinDA—demonstrated high statistical power (**Figure 2**) when the sample size was large (e.g., *n* = 80 or *n* = 160), suggesting that each method can reliably detect true effects. However, in small-sample settings, GLM-ASCA exhibited greater power than MaAsLin2 and LinDA, indicating improved performance with limited sample sizes. This improved performance is likely due to its multivariate modeling framework, which captures shared patterns across features and leverages the joint data structure to detect effects even with few samples. Despite this difference in power, all methods performed comparably on measures such as specificity, AUC, F1-score, and MCC. Moreover, all three methods effectively maintained FDR control at the nominal 5% level.

The low feature–feature correlations observed in the template plant microbiome data (**Supplementary Figure S15**), particularly for large sample sizes, appear to favor MaAsLin2 and LinDA, since these univariate methods perform optimally when features are weakly dependent and sufficient data support stable parameter estimation. Taken together, these findings suggest that while MaAsLin2 and LinDA are robust choices for univariate analysis in large-sample contexts, GLM-ASCA offers a notable advantage in small-sample, structured experimental designs by leveraging multivariate information.

**Figure 3:**
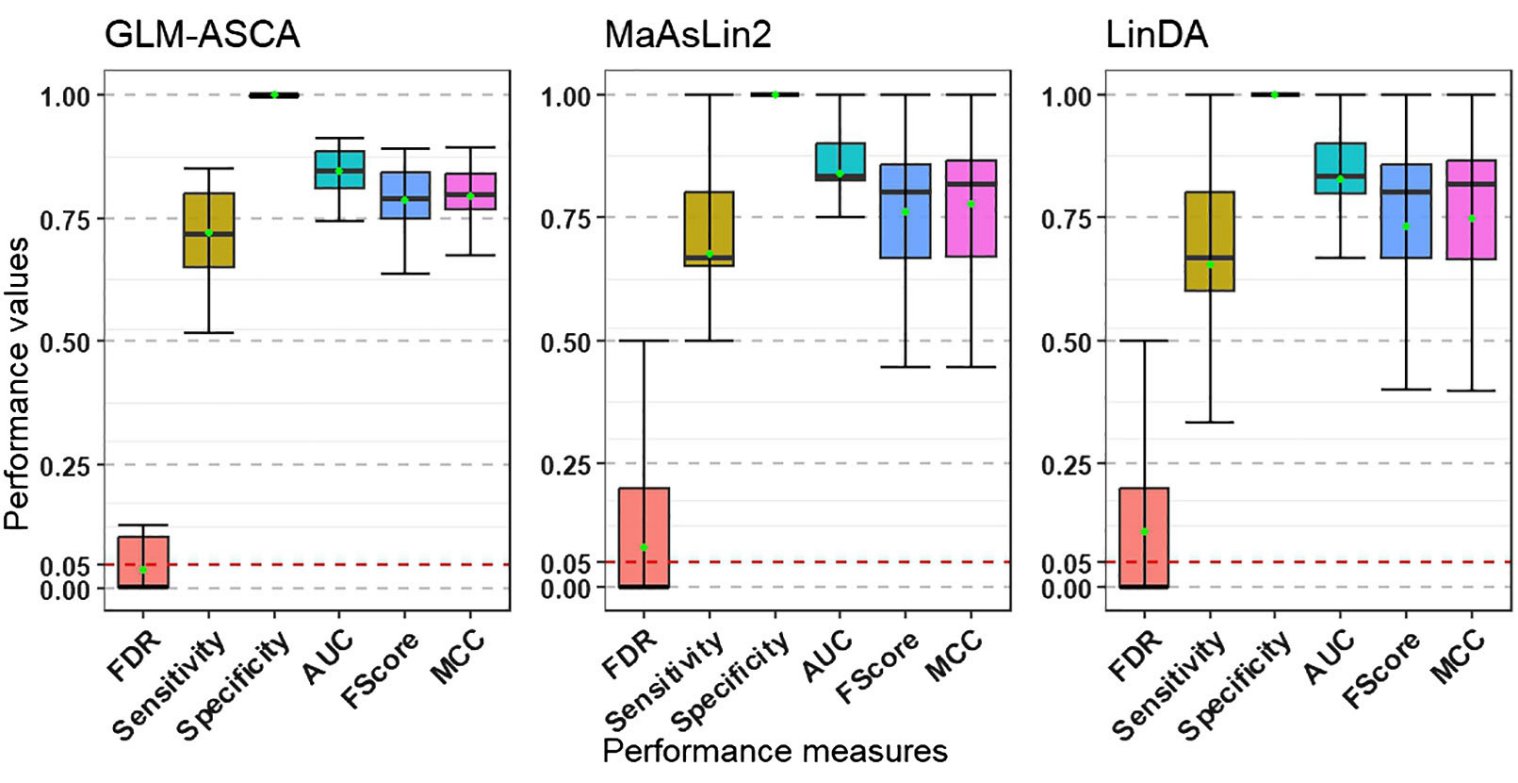
Performance measures (FDR, statistical power [sensitivity], specificity, AUC, F1-score, and MCC) for GLM–ASCA, MaAsLin2, and LinDA, evaluated on simulated data derived from a template plant microbiome dataset with 40 samples, 1000 features, and varying sparsity. Boxplots are colored by performance measures. Compared with MaAsLin2 and LinDA, GLM-ASCA demonstrated mean FDR (green dots) close to the nominal 5% level while maintaining moderately high statistical power.

We extended the simulation study using SparseDOSSA2 by increasing the number of features from 206 to 1000 with varying sparsity (proportion of zeros), while retaining the small-sample scenario (*n* = 40) and the same data generation procedure. This reflects common challenges in microbiome research, where datasets often involve limited samples and many sparse features. As shown in **Figure 3**, GLM-ASCA maintained robust performance under these conditions, effectively controlling FDR at the nominal 5% level while achieving moderately high statistical power. These findings highlight the effectiveness of GLM-ASCA in detecting true features and controlling false discoveries despite high dimensionality and small sample sizes. In terms of AUC, F1-score, and MCC, all three methods showed comparable performance in this small-sample scenario.

### 3.2 Microbiome data analysis results

The Tweedie-based GLM-ASCA was applied to tomato root microbiome data to identify microbes whose abundance was significantly affected by nitrogen starvation over time. The design matrix included growth condition with two levels (N-starvation: nitrogen starved; control: nitrogen rich), time with four levels (4, 8, 12, and 16 days), and their interaction (growth condition × days). The following generalized linear model was used to estimate the effect matrices:


(19) $$log(\mu )=\beta_{0}+\beta_{G}\cdot \text{GrowthCondition}+\beta_{D}\cdot \text{Days}+\beta_{GD}\cdot \text{GrowthCondition}\;\times \;\text{Days},$$

with a logarithm link function relating mean microbial abundance to the experimental factors. Normalized counts were computed using *poscounts* normalization from the R package DESeq2. The design matrix was coded using sum coding. Out of 5300 ASVs, 1009 ASVs were retained after filtering for a minimum of 5 counts in at least 3 of the 5 replicates in each growth condition–time combination. The design was balanced, comprising 40 samples with 5 replicates for each condition and time point, and the GLM model (Equation 19) was saturated, including all main and interaction effects. Thus, two basic requirements for GLM-ASCA were satisfied: balanced design and saturated model specification.

For each filtered feature, univariate GLMs were fitted using the Tweedie distribution with the R packages tweedieverse and mcglm, which allow estimation of the dispersion (*ϕ*), power (*ρ*), and regression parameters (*β*). Estimates of the Tweedie power and dispersion parameters are shown in **Supplementary Figure S3**. The regression parameter estimates $\left(\hat{\beta }\right)$ and design matrix were then used to calculate the effect matrices for the main effects of growth condition and time, as well as their interaction. PCA was applied to each effect matrix to obtain the score and loading matrices. **Table 1** displays the percentages of explained variation due to main and interaction effects, calculated based on the adjusted response. The experimental conditions accounted for ~88% of the total variation. **Table 1** also includes p-values from global tests of effects, computed using the Frobenius norm of principal component score matrices (see Section 2.1.3), which revealed significant main and interaction effects (*p <* 0.05).

**Table 1:** Percentage of explained variation in the adjusted abundance response of the tomato microbiome data, accounted for by experimental factors, using the Tweedie GLM-ASCA model.

Component	Explained variation (%)	Permutation p-values
Growth Condition	21.99	0.0001
Days	34.89	0.0001
Growth Condition x Days	30.87	0.0005
Residuals	12.25	
Total	100.00	

**Figure 4:**
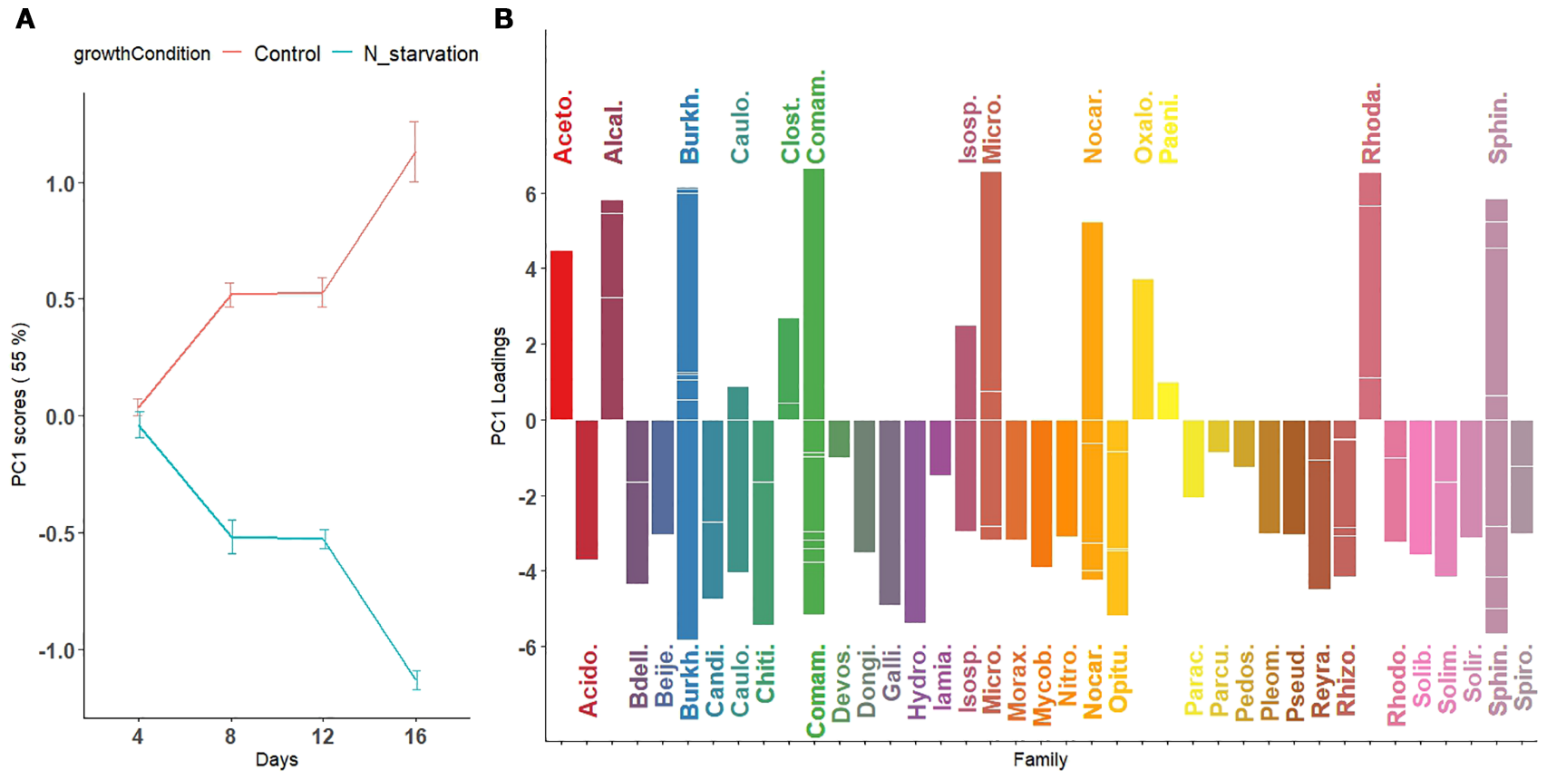
First principal component visualizing temporal variation in microbial abundance patterns across taxa under nitrogen starvation and control conditions. **(A)** Scores of the first principal component are plotted for nitrogen-starved (green line) and nitrogen-rich (control; red line) conditions over time (days). The trajectories represent overall trends in microbial abundance under each condition. **(B)** Loadings of the first principal component, representing the contribution of individual microbial species, are displayed as bars colored by their bacterial families. Downward-facing bars correspond to families with higher relative abundance under nitrogen-starved conditions (green trajectory in panel A), whereas upward-facing bars correspond to families with higher relative abundance under nitrogen-rich (control) conditions (red trajectory in panel A). For microbial families with multiple contributing species, individual species loadings are indicated by horizontal segments within the same vertical bar.

**Figure 5:**
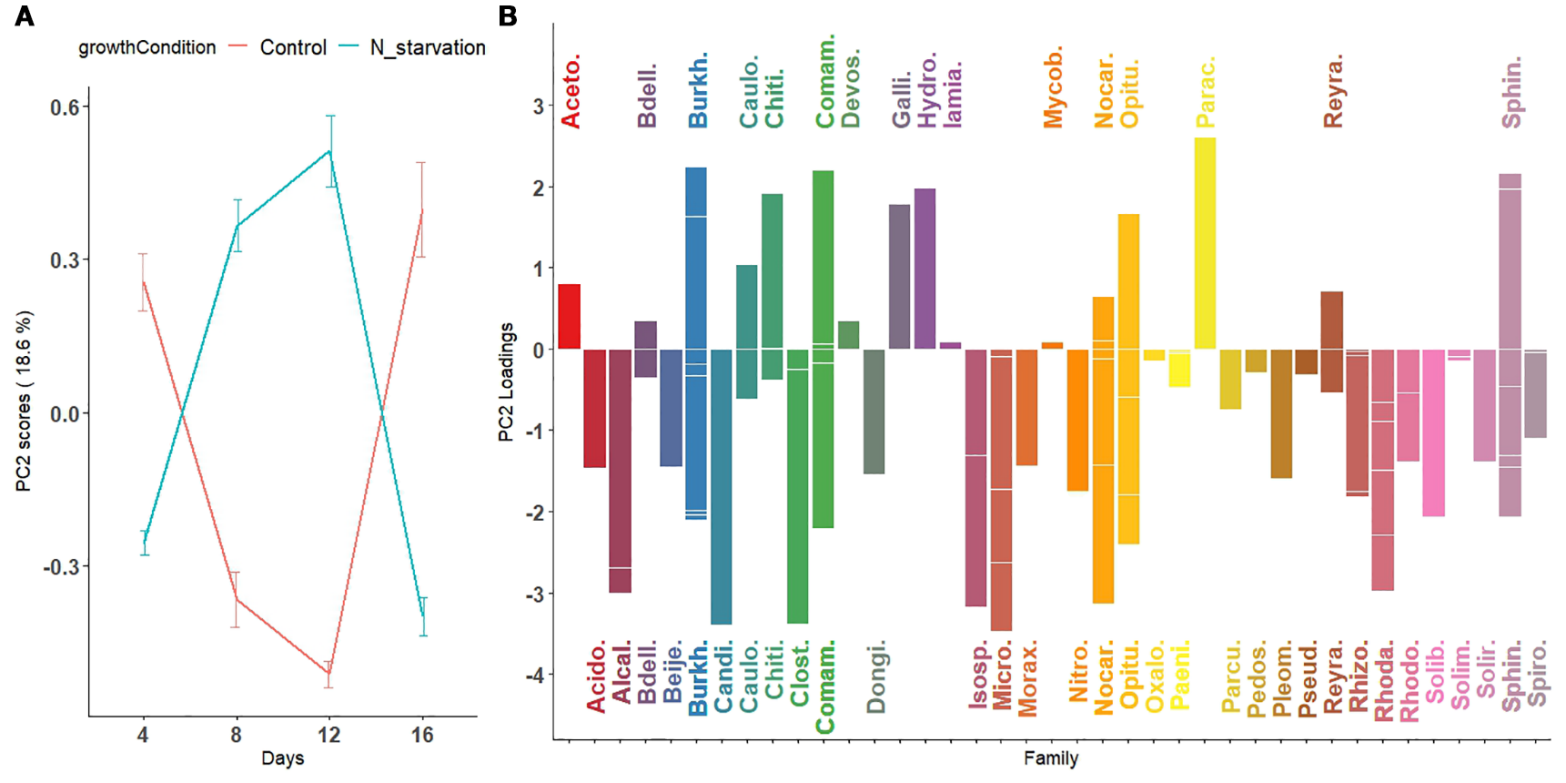
Second principal component visualizing nonlinear temporal patterns in microbial abundance across taxa under nitrogen starvation and control conditions. **(A)** Scores of the second principal component reveal a nonlinear pattern characterized by either a peak (for taxa with positive loadings) or a dip (for taxa with negative loadings) around day 12 in both nitrogen-starved and nitrogen-rich (control) conditions. **(B)** Loadings of the second principal component, representing the contribution of individual microbial species to this nonlinear pattern, are displayed as bars colored by their bacterial families. These trajectories capture temporal fluctuations in microbial abundance not explained by the first principal component, highlighting complex, taxa-specific responses to nitrogen availability throughout the experimental period.

One advantage of ASCA-based approaches is the ability to visualize effects using score and loading matrices. Results are shown in **Supplementary Figures S8**-**S14**; **Figures 4**, **5**. In these figures, points represent principal component scores computed for the two growth conditions at each time point. Lines connect scores across successive time points to illustrate temporal dynamics in microbial relative abundance. Error bars correspond to mean ± 1 standard deviation of the projected scores (Equation 6).

Because of the significant interaction effect between time and growth condition, the main effects (**Supplementary Figures S8**-**S14**) should not be interpreted separately. Thus, we combined the main effect of growth condition with the interaction effect of time (GrowthCondition + GrowthCondition × Days). This, in particular, allows for a more direct assessment of how the microbial abundance in the control and N-starvation groups changes over time during tomato growth. We applied PCA to the combined effect matrix (GrowthCondition + GrowthCondition × Days). Using the scaled leverage permutation test with 10,000 permutations, 121 ASVs that belong to 44 families were identified (**Figure 4B**, **Supplementary Figure S4**) to be significantly affected by nitrogen starvation over time (adjusted p-value *<* 0.05). The first two principal components from the combined effect matrix (**Figures 4**, **5**) accounted for 75.3% of the total variation. On the first principal component, no significant differences were observed between the average principal scores of the N-starvation and control groups at day 4. However, beyond day 4, thedifference between groups increased over time (**Figure 4A**), indicating increasing divergence in microbial abundance between nitrogen-starved and nitrogen-rich conditions.

From loading plots (**Figures 4B**, **Supplementary Figures S4**, **S5**), families with positive loadings in the control group showed increasing microbial abundance over time, whereas in the N-starvation group, families with negative loadings showed increasing abundance. Families/genera enriched under nitrogen starvation included Acidobacteriaceae (Paludibaculum), Bdellovibrionaceae (Bedellovibrio), Burkholderiaceae (Polynucleobator), Caulobactraceae (Asticcacaulis), Chitinophagaceae (Terrimonas and Edaphobaculum), Comamonadaceae (Acidovorax, Aquabacterium and Methylibium), Gallionellacea (Candidatus Nitrotoga), Hydrogenophilaceae (Thiobacillus), Mycobacteriaceae (Mycobacterium), Nocardioidaceae (Aeromicrobium and Nocardioides), Opitutaceae (Opitutus), Pleomorphomonadaceae (Pleomorphomonas), Pseudomonadaceae (Pseudomonas), Reyranellaceae (Reyranella), Rhizobiaceae (AllorhizobiumNeorhizobiumPararhizobiumRhizobium, and Mesorhizobium), Solimonadaceae (Solimonas), and Sphingomonadaceae (Novosphingobium, Sphingobium and Sphingomonas) include one or more species that showed significantly increased abundance under nitrogen starvation, whereas families (genera) such as: Acetobacteraceae (Acidisoma), Alcaligenaceae (Bordetella), Burkholderiaceae (Burkholderia-CaballeroniaParaburkholderia, Robbsia and Pandoraea), Clostridiaceae (Clostridium), Comamonadaceae (Thiomonas), Microbacteriaceae (Leifsonia), Oxalobacteraceae, Rhodanobacteraceae (Rhodanobacter), and Sphingobacteriaceae (Mucilaginibacter) include one or more species that showed a significant increase in abundance under the control or nitrogen availability condition. When multiple species contributed significantly, loadings are represented by horizontal bars within each family.

In the second principal component (**Figure 5**), average scores followed a nonlinear (parabolic) pattern of microbial abundance over time. Under N-starvation, average abundance of Gallionellaceae, Hydrogenophilaceae, and Parachlamydiaceae increased until day 12, then sharply declined at day 16. Similarly, under control conditions, Alcaligenaceae, Candidatus Kaiserbacteria, Clostridiaceae, Microbacteriaceae, and Rhodanobacteraceae exhibited such curved abundance profiles. The third and fourth principal components, explaining ~26% of the remaining variation, are shown in **Supplementary Figures S6**-**S7**.

Enrichment of bacterial genera under nitrogen starvation highlights their potential roles in adapting to and mitigating nitrogen limitation. Several taxa identified here have previously been reported in nitrogen-related processes. For instance, species within Terrimonas (Guo et al., 2024), Thiobacillus (Li et al., 2023), Mycobacterium (Sellstedt and Richau, 2013), Pseudomonas (Wu et al., 2023; Sanow et al., 2023), Sphingomonas (Videira et al., 2009), Novosphingobium (Addison et al., 2007), Mesorhizobium (Menéndez et al., 2020), and Allorhizobium*–*Neorhizobium*–*Pararhizobium*–*Rhizobium (You et al., 2021) are known nitrogen fixers, with *nifH* genes detected in many studies. Similarly, Candidatus Nitrotoga (Lucker et al., 2015; Boddicker and Mosier, 2018), Aquabacterium (Zhang et al., 2016a), and Sphingobium (Boss et al., 2022) are implicated in nitrogen cycling. These taxa may enhance nitrogen availability to the plant either by directly fixing atmospheric nitrogen into plant-available forms such as ammonium, by contributing to nitrogen mineralization processes that convert organic nitrogen compounds into inorganic forms like ammonium and nitrate (Philippot et al., 2013), or by adapting plant development, such as root architecture (Abedini et al., manuscript in preparation^1^).

We also observed an increase in Bdellovibrio (Bratanis et al., 2020), a potential biocontrol agent. Increased Bdellovibrio abundance may reflect a regulatory mechanism suppressing pathogenic or competing bacteria, indirectly supporting beneficial taxa and plant health.

To validate our findings, for example, Sphingobium was identified as enriched under nitrogen deficiency. Isolation of a Sphingobium strain from nitrogen-starved tomato roots, followed by *in vitro* assays, confirmed that it can stimulate tomato growth under nitrogen-deficient conditions (Abedini et al., manuscript in preparation^1^). These findings suggest that nitrogen deficiency alters microbial community structure, and that recruited taxa support plant adaptation by enhancing nitrogen availability. Overall, the results highlight the effectiveness of GLM-ASCA in identifying key microbial taxa under specific experimental conditions, underscoring its potential as a powerful tool for microbiome data analysis.

## 4 Discussion

Statistical analysis of high-dimensional, non-normal, and non-linear data—such as those obtained from microbiome studies—and incorporating experimental design elements such as treatments, time, and interactions present challenges because traditional statistical methods often assume normality and may not be appropriate for such datasets. Advanced statistical tools, such as ANOVA simultaneous component analysis (ASCA/ASCA+), have emerged as valuable approaches, providing insights into the main sources of variability and facilitating interpretation. However, adapting ASCA to count data with excess zeros, such as microbiome data, necessitates novel approaches. This led us to develop GLM-ASCA (generalized linear models–ANOVA simultaneous component analysis), which integrates treatment design elements and GLMs within a multivariate framework.

The simulation results demonstrated effective control over false discovery rates, highlighting the potential of GLM-ASCA as a robust feature selection tool. Application of GLM-ASCA to microbiome data to assess the effect of nitrogen starvation on tomato over time identified several bacterial families and genera that exhibited increased abundance under nitrogen deficiency, many of which have been implicated in nitrogen metabolism in previous studies. The observed changes in microbial abundance during nitrogen starvation suggest that plants modulate root exudation patterns to selectively recruit beneficial microbial taxa. These microbes contribute to nitrogen availability and support plant growth through multiple complementary mechanisms, including nitrogen cycling and mineralization, symbiotic and free-living nitrogen fixation, root colonization coupled with plant growth promotion, stress adaptation and stabilization of the rhizosphere under nutrient-limited conditions, and microbial community regulation and niche structuring.

For instance, the increased abundance of genera such as Sphingobium, Pseudomonas, and Mesorhizobium suggests potential mechanisms where these microbes enhance nitrogen availability either through biological nitrogen fixation or mineralization pathways. To further support and validate these results, whole-genome sequencing and co-culturing assays were conducted with Sphingobium sp. RS1, a strain isolated from nitrogen-starved tomato roots. These analyses revealed several plant growth–promoting traits, including the production of phytohormones such as indole-3-acetic acid (IAA) and the ability to mineralize organic nitrogen into plant-available forms (Abedini et al., manuscript in preparation^1^). Although this targeted validation experiment supported the role of specific taxa such as Sphingobium, many other microbial taxa identified in the present study as potentially involved in mitigating nitrogen deficiency require further validation. Rigorous experimental confirmation is necessary to determine their functional roles and assess their effectiveness in tomato, a nonlegume crop, under both controlled and field conditions. Once thoroughly validated, these results could enable the development of targeted inoculants or synthetic microbial consortia designed to improve plant growth and health in nitrogen-limited environments. Ultimately, such bio-based strategies have the potential to support sustainable agriculture by reducing dependence on chemical fertilizers and promoting more efficient nutrient use in crop production systems.

The results from both simulated and real data underscore the utility of the GLM-ASCA framework as an effective tool for identifying key microbial species responding to specific experimental conditions, treatments, or interactions. However, a crucial aspect of the current development of GLM-ASCA is its reliance on data from balanced experimental designs with model specifications that include all main and interaction effects. We are currently working on expanding the framework by incorporating different link functions and extending it to more general scenarios, including balanced designs without specification constraints and unbalanced designs.

Finally, with the growing importance of plant microbiome research in sustainable agriculture and human health, developing such statistical tools is crucial for identifying biologically important microbes that play key roles in enhancing agricultural practices and improving health outcomes. Moreover, the ability of GLM-ASCA to effectively handle complex experimental designs and accurately analyze microbial abundance patterns highlights its potential for broader applications beyond plant microbiome research. GLM-ASCA can be applied in various fields that require the analysis of high-dimensional, compositional, and zero-inflated data with complex experimental designs, including human microbiome studies, other omics applications involving high-throughput sequencing, and ecological studies.

## Data Availability

The original contributions presented in the study are included in the article/**Supplementary Material**. Further inquiries can be directed to the corresponding author. All data sets generated and analyzed and the R-code used to analyze the data are available in Figshare public repository https://figshare.com/s/744f99a21afca4d6c002.

## Footnotes

1 Abedini, D., White, F., Jain, R., Guerrieri, A., Schram, R., Dong, L., et al. (2025). Multi-omics data analysis revealed a novel beneficial role for strigolactones in tomato.
