# Supplementary material for: Analysis of microbiome high-dimensional experimental design data using generalized linear models and ANOVA simultaneous component analysis
Source: Front Microbiomes. 2025 Oct 15;4:1584516. doi: 10.3389/frmbi.2025.1584516 (PMC12993487; doi:10.3389/frmbi.2025.1584516)
Supplement: Supplementary file 1 [file DataSheet1.pdf]

Supplementary Material for Analyzing Microbiome High Dimensional Experimental Design Data using Generalized Linear Models and ANOVA Simultaneous Component Analysis

1 SUPPLEMENTARY DATA

Text S1: Generalzed linear models (GLMs)

In generalized linear models (McCullagh and Nelder, 1989), three main components are involved in linking the response variable Y to the design matrix $\mathbf{X}$. The first component is related to the assumption that the distribution of each element of the response variable Y follows independent exponential family of distributions given by

$$f(y;\theta,\phi) = \exp\left(\frac{y\theta - b(\theta)}{a(\phi)} + c(y,\phi)\right), \tag{S1}$$

where θ is called the canonical parameter and represents the location, while ϕ is called the dispersion parameter and represents the scale, and $a(\cdot)$, $b(\cdot)$ and $c(\cdot)$ are continuous functions. The mean of Y is given by $\mu = E[Y] = b'(\theta) = db(\theta)/d\theta$ and the variance of Y is $\text{Var}(Y) = b''(\theta)a(\phi) = a(\phi)V(\mu)$, where $V(\mu) = b''(\theta) = d\mu/d\theta$ is called the variance function that describes the relationship between variance and mean.

The second component is the linear predictor, for the i-th observation,

$$\eta_i = \beta_0 + \beta_1 x_{i1} + \cdots + \beta_p x_{ik} = \mathbf{x_i}^T\beta,$$

where $x_i = (x_{i0}, x_{i1}, \ldots, x_{ik})$ is the i-th row of $\mathbf{X}$ and $x_{i0} = 1$, $i = 1, \ldots, n$. The unknown regression parameters, $\beta = (\beta_0, \beta_1, \ldots, \ldots, \beta_k)^T$ need to be estimated from data. In matrix form, $\eta = \mathbf{X}\beta$.

The third component is the link function g that relates the mean to the linear predictor

$$\eta_i = g(\mu_i).$$

Although the link function g can be any monotone and differentiable function, some convenient and common choices are preferred for GLMs. In particular, choosing the canonical link function g such that $\eta = g(\mu) = \theta$ in (S1) simplifies the derivation of the maximum likelihood estimation and ensures that μ remains within the range of the response variable Y. For example, the logarithm and logistic canonical link functions are commonly used in Poisson and binomial regressions, respectively.

In the GLM setting, for convenience, the dependent variable is not the observed $\mathbf{y}$ but a linearized form of the link function applied to $\mathbf{y}$, called the working response denoted by $\mathbf{z}$. For the i-th observation, the working response is defined as $z_i = \eta_i + \frac{\partial \eta_i}{\partial \mu_i}(y_i - \mu_i)$. Similarly, the following notations are also used for

the i-th linear predictor $\eta_i = \sum_{j=0}^{k} x_{ij}\beta_j$, mean $\mu_i = g^{-1}(\eta_i)$, weights $w_i = \left(\frac{\partial\mu_i}{\partial\eta_i}\right)^2 / V(\mu_i)$ and variance function $V(\mu_i)$. Denote by $\mathbf{W}$ a diagonal matrix of weights $w_{ii},\ \ i = 1, \ldots, n$.

The maximum likelihood estimators for β are derived through iterative algorithms like the Newton-Raphson method or the iteratively reweighted least squares (IRLS) method (McCullagh and Nelder, 1989). At each iteration until convergence, $z_i,\ w_i,\ \beta,\ \eta_i$ and μ_i are updated. Once the IRLS algorithm has converged, the maximum likelihood estimate of β is given by

$$\hat{\beta} = (\mathbf{X}^T\hat{\mathbf{W}}\mathbf{X})^{-1}\mathbf{X}^T\hat{\mathbf{W}}\hat{\mathbf{z}}.$$

The estimated $\hat{\beta}$ can be used to obtain the final maximum likelihood estimates $\hat{\eta} = \mathbf{X}\hat{\beta}$, $\hat{\mu} = g^{-1}(\hat{\eta})$, $\hat{\mathbf{W}}$ a diagonal matrix of estimated weights $\hat{w}_i = \left(\frac{\partial\mu_i}{\partial\eta_i}\right)^2 |_{\hat{\mu}_i} / V(\hat{\mu}_i)$ and the working response $\hat{\mathbf{z}}$, with $\hat{z}_i = \hat{\eta}_i + \left(\frac{\partial\eta_i}{\partial\mu_i}\right) |_{\hat{\eta}_i}(y_i - \hat{\mu}_i)$.

One of the residuals in GLM called working residuals is obtained by

$$\hat{\mathbf{r}}^w = \hat{\mathbf{z}} - \hat{\eta}.$$

Moreover, similar to linear models, the Hat matrix which in generalized linear models is given by

$$\hat{\mathbf{H}} = \hat{\mathbf{W}}^{1/2}\mathbf{X}(\mathbf{X}^T\hat{\mathbf{W}}\mathbf{X})^{-1}\mathbf{X}^T\hat{\mathbf{W}}^{1/2}, \tag{S2}$$

is idempotent and symmetric.

Similarities between linear models and generalized linear models

Even though the similarity between linear modes (LMs) and generalized linear models (GLMs) has long been recognized, Lovison (2014) refined this similarity by systematizing and confirming several findings that are either dispersed or inadequately described in the literature. The vast majority of theory underlying GLMs comes from an effort to extend the rich body of methods developed for LMs to situations in which the standard assumptions of LMs are violated: non-linear relationship, absence of homoscedasticity, and non-normality (Lovison, 2014). The implementation of the IRLS algorithm, in particular, was critical in establishing similarities and differences between GLMs and LMs. For example, the closest counterparts of response observations (y), fitted values $(\hat{y})$ and ordinary residuals $(r = y - \hat{y})$ in LMs are represented by the working response values $(\hat{z})$, the estimated linear predictor $(\hat{\eta})$ and the working residuals $(\hat{r}^w = \hat{z} - \hat{\eta})$ in GLMs(Lovison, 2014). Moreover, using scaled versions of covariates $(\hat{\mathbf{W}}^{1/2}\mathbf{X})$, linear predictor $\hat{\eta}^* = \hat{\mathbf{W}}^{1/2}\hat{\eta}$, working response $\hat{\mathbf{z}}^* = \hat{\mathbf{W}}^{1/2}\hat{\mathbf{z}}$, and working residuals $\hat{\mathbf{r}}^{w*} = \hat{\mathbf{W}}^{1/2}\hat{\mathbf{r}}^w$ as defined by Lovison help to demonstrate the similarities of many GLM and LM results(see Table S1 below).

Text S2: Examples of balanced designs and saturated models

In a 2×2 factorial design with a saturated model that includes the overall mean (intercept term), two main factors, and their interaction, the design data $(\mathbf{X} = \mathbf{X}_{2\times2}^{(K)})$, K indicating the number of replicates) can be expressed using the four parameters $(\mu,\ \ \alpha,\ \ \beta,\ \ (\alpha\beta))$ and $n = 4$ experimental units based on sum coding for one replication $(K = 1)$ per experimental unit as

$$\mathbf{X}^{(1)}_{2\times 2} = \begin{array}{c} \\ O_1 \\ O_2 \\ O_3 \\ O_4 \end{array} \begin{array}{c} \begin{array}{cccc} X_\mu & X_\alpha & X_\beta & X_{(\alpha\beta)} \end{array} \\ \begin{pmatrix} 1 & 1 & 1 & 1 \\ 1 & 1 & -1 & -1 \\ 1 & -1 & 1 & -1 \\ 1 & -1 & -1 & 1 \end{pmatrix} \end{array},$$

where O_i denotes experimental units or observations. Similarly, the weight matrix is given by a diagonal matrix of GLM weights

$$\hat{\mathbf{W}}_n = \begin{pmatrix} w_1 & 0 & 0 & 0 \\ 0 & w_2 & 0 & 0 \\ 0 & 0 & w_3 & 0 \\ 0 & 0 & 0 & w_4 \end{pmatrix}.$$

Similarly, for a 3×2 factorial design with a saturated model that includes the overall mean, two main effects for the first factor with 3 levels, one main effect term for the second factor with 2 levels, and two interaction terms, the design data $(\mathbf{X} = \mathbf{X}^{(K)}_{3\times 2})$ can be coded with 6 parameters and 6 experimental units using sum coding for one replication $(K = 1)$ per experimental unit as

$$\mathbf{X}^{(1)}_{3\times 2} = \begin{array}{c} \\ O_1 \\ O_2 \\ O_3 \\ O_4 \\ O_5 \\ O_6 \end{array} \begin{array}{c} \begin{array}{cccccc} X_\mu & X_{\alpha_1} & X_{\alpha_2} & X_\beta & X_{(\alpha_1\beta)} & X_{(\alpha_2\beta)} \end{array} \\ \begin{pmatrix} 1 & 1 & 0 & 1 & 1 & 0 \\ 1 & 1 & 0 & -1 & -1 & 0 \\ 1 & 0 & 1 & 1 & 0 & 1 \\ 1 & 0 & 1 & -1 & 0 & -1 \\ 1 & -1 & -1 & 1 & -1 & -1 \\ 1 & -1 & -1 & -1 & 1 & 1 \end{pmatrix} \end{array},$$

and GLM based weights

$$\hat{\mathbf{W}}_n = \begin{pmatrix} w_1 & 0 & 0 & 0 & 0 & 0 \\ 0 & w_2 & 0 & 0 & 0 & 0 \\ 0 & 0 & w_3 & 0 & 0 & 0 \\ 0 & 0 & 0 & w_4 & 0 & 0 \\ 0 & 0 & 0 & 0 & w_5 & 0 \\ 0 & 0 & 0 & 0 & 0 & w_6 \end{pmatrix}.$$

In general, for $K > 1$ replications included in the experiment, the data structure can be expressed by vertically concatenating the $X^{(1)}$ coding matrices as

$$\mathbf{X} = \begin{array}{c} B_1 \\ B_2 \\ \vdots \\ B_K \end{array} \begin{pmatrix} \mathbf{X}^{(1)} \\ \mathbf{X}^{(1)} \\ \vdots \\ \mathbf{X}^{(1)} \end{pmatrix}, \tag{S3}$$

where B_i is used to indicate a block of n experimental units for the i-th replication. The weight matrix is given as a block diagonal matrix

$$\hat{\mathbf{W}} = \begin{matrix} B_1 \\ B_2 \\ \vdots \\ B_K \end{matrix} \begin{pmatrix} \hat{\mathbf{W}}_n & 0 & \cdots & 0 \\ 0 & \hat{\mathbf{W}}_n & \cdots & 0 \\ \vdots & \vdots & \ddots & \vdots \\ 0 & 0 & \cdots & \hat{\mathbf{W}}_n \end{pmatrix}. \qquad \text{(S4)}$$

Text S3: Hat matrix derivation for balanced and saturated designs in GLMs

Using equations (S3) and (S4) and letting

$$\mathbf{Q} = \hat{\mathbf{W}}^{1/2}\mathbf{X} = \begin{pmatrix} \hat{\mathbf{W}}_n^{1/2}\mathbf{X}^{(1)} \\ \hat{\mathbf{W}}_n^{1/2}\mathbf{X}^{(1)} \\ \vdots \\ \hat{\mathbf{W}}_n^{1/2}\mathbf{X}^{(1)} \end{pmatrix},$$

the Hat matrix (S2) is rewritten as

$$\hat{\mathbf{H}} = \mathbf{Q}\left(\mathbf{Q}^T\mathbf{Q}\right)^{-1}\mathbf{Q}^T,$$

where

$$\mathbf{Q}^T\mathbf{Q} = \mathbf{X}^{(1)T}\hat{\mathbf{W}}_n\mathbf{X}^{(1)} + \cdots + \mathbf{X}^{(1)T}\hat{\mathbf{W}}_n\mathbf{X}^{(1)} = K \cdot \mathbf{X}^{(1)T}\hat{\mathbf{W}}_n\mathbf{X}^{(1)},$$

and its inverse

$$\left(\mathbf{Q}^T\mathbf{Q}\right)^{-1} = \frac{1}{K}\left(\mathbf{X}^{(1)T}\hat{\mathbf{W}}_n\mathbf{X}^{(1)}\right)^{-1}.$$

Then the Hat matix is

$$\hat{\mathbf{H}} = \begin{pmatrix} \hat{\mathbf{W}}_n^{1/2}\mathbf{X}^{(1)} \\ \hat{\mathbf{W}}_n^{1/2}\mathbf{X}^{(1)} \\ \vdots \\ \hat{\mathbf{W}}_n^{1/2}\mathbf{X}^{(1)} \end{pmatrix} \times \left(\frac{1}{K}\left(\mathbf{X}^{(1)T}\hat{\mathbf{W}}_n\mathbf{X}^{(1)}\right)^{-1}\right) \times \left(\mathbf{X}^{(1)T}\hat{\mathbf{W}}_n^{1/2}, \ldots, \mathbf{X}^{(1)T}\hat{\mathbf{W}}_n^{1/2}\right)$$

$$\hat{\mathbf{H}} = \begin{pmatrix} \frac{1}{K}\hat{\mathbf{W}}_n^{1/2}\mathbf{X}^{(1)}\left(\mathbf{X}^{(1)T}\hat{\mathbf{W}}_n\mathbf{X}^{(1)}\right)^{-1}\mathbf{X}^{(1)T}\hat{\mathbf{W}}_n^{1/2} & \cdots & \frac{1}{K}\hat{\mathbf{W}}_n^{1/2}\mathbf{X}^{(1)}\left(\mathbf{X}^{(1)T}\hat{\mathbf{W}}_n\mathbf{X}^{(1)}\right)^{-1}\mathbf{X}^{(1)T}\hat{\mathbf{W}}_n^{1/2} \\ \vdots & \cdots & \vdots \\ \frac{1}{K}\hat{\mathbf{W}}_n^{1/2}\mathbf{X}^{(1)}\left(\mathbf{X}^{(1)T}\hat{\mathbf{W}}_n\mathbf{X}^{(1)}\right)^{-1}\mathbf{X}^{(1)T}\hat{\mathbf{W}}_n^{1/2} & \cdots & \frac{1}{K}\hat{\mathbf{W}}_n^{1/2}\mathbf{X}^{(1)}\left(\mathbf{X}^{(1)T}\hat{\mathbf{W}}_n\mathbf{X}^{(1)}\right)^{-1}\mathbf{X}^{(1)T}\hat{\mathbf{W}}_n^{1/2} \end{pmatrix},$$

and using equation (11) for each replication $k = 1, \ldots, K$,

$$\hat{\mathbf{W}}_n^{1/2}\mathbf{X}^{(k)}\left(\mathbf{X}^{(k)T}\hat{\mathbf{W}}_n\mathbf{X}^{(k)}\right)^{-1}\mathbf{X}^{(k)T}\hat{\mathbf{W}}_n^{1/2} = \mathbf{I}_{n\times n},$$

it follows that

$$\hat{\mathbf{H}} = \begin{pmatrix} \frac{1}{K}\mathbf{I}_{n\times n} & \cdots & \frac{1}{K}\mathbf{I}_{n\times n} \\ \vdots & & \vdots \\ \frac{1}{K}\mathbf{I}_{n\times n} & \cdots & \frac{1}{K}\mathbf{I}_{n\times n} \end{pmatrix}.$$

Text S4: Examples of Hat matrices in balanced and saturated designs in GLMs

Example1. For the 2×2 factorial design with $K = 2$ replicates and 4 experimental units using a saturated model, the Hat matrix can be expressed in two ways. (1) Using experimental units based arrangement of replications (i.e., putting replications under each experimental unit), and (2) Using a replication-wise arrangement of experimental units (i.e., putting experimental units under each replication).

The design matrix and the corresponding Hat matrix using experimental units based arrangement of replications as in (S3) are given by

$$\mathbf{X} = \left(\begin{array}{cccc} 1 & 1 & 1 & 1 \\ 1 & 1 & -1 & -1 \\ 1 & -1 & 1 & -1 \\ 1 & -1 & -1 & 1 \\ \hline 1 & 1 & 1 & 1 \\ 1 & 1 & -1 & -1 \\ 1 & -1 & 1 & -1 \\ 1 & -1 & -1 & 1 \end{array}\right), \quad \hat{\mathbf{H}} = \left(\begin{array}{cccc|cccc} \frac{1}{2} & 0 & 0 & 0 & \frac{1}{2} & 0 & 0 & 0 \\ 0 & \frac{1}{2} & 0 & 0 & 0 & \frac{1}{2} & 0 & 0 \\ 0 & 0 & \frac{1}{2} & 0 & 0 & 0 & \frac{1}{2} & 0 \\ 0 & 0 & 0 & \frac{1}{2} & 0 & 0 & 0 & \frac{1}{2} \\ \hline \frac{1}{2} & 0 & 0 & 0 & \frac{1}{2} & 0 & 0 & 0 \\ 0 & \frac{1}{2} & 0 & 0 & 0 & \frac{1}{2} & 0 & 0 \\ 0 & 0 & \frac{1}{2} & 0 & 0 & 0 & \frac{1}{2} & 0 \\ 0 & 0 & 0 & \frac{1}{2} & 0 & 0 & 0 & \frac{1}{2} \end{array}\right) = \frac{1}{2}\cdot\left(\begin{array}{cccc|cccc} 1 & 0 & 0 & 0 & 1 & 0 & 0 & 0 \\ 0 & 1 & 0 & 0 & 0 & 1 & 0 & 0 \\ 0 & 0 & 1 & 0 & 0 & 0 & 1 & 0 \\ 0 & 0 & 0 & 1 & 0 & 0 & 0 & 1 \\ \hline 1 & 0 & 0 & 0 & 1 & 0 & 0 & 0 \\ 0 & 1 & 0 & 0 & 0 & 1 & 0 & 0 \\ 0 & 0 & 1 & 0 & 0 & 0 & 1 & 0 \\ 0 & 0 & 0 & 1 & 0 & 0 & 0 & 1 \end{array}\right)$$

Similar Hat matrix structures are also described for ANOVA fixed effect models (Orenti et al., 2012).

Similarly, using experimental units based arrangement of replications, the design matrix and the corresponding Hat matrix can be expressed as

$$\mathbf{X} = \left(\begin{array}{cccc} 1 & 1 & 1 & 1 \\ 1 & 1 & 1 & 1 \\ \hline 1 & 1 & -1 & -1 \\ 1 & 1 & -1 & -1 \\ \hline 1 & -1 & 1 & -1 \\ 1 & -1 & 1 & -1 \\ \hline 1 & -1 & -1 & 1 \\ 1 & -1 & -1 & 1 \end{array}\right), \quad \hat{\mathbf{H}} = \begin{pmatrix} \frac{1}{2} & \frac{1}{2} & 0 & 0 & 0 & 0 & 0 & 0 \\ \frac{1}{2} & \frac{1}{2} & 0 & 0 & 0 & 0 & 0 & 0 \\ 0 & 0 & \frac{1}{2} & \frac{1}{2} & 0 & 0 & 0 & 0 \\ 0 & 0 & \frac{1}{2} & \frac{1}{2} & 0 & 0 & 0 & 0 \\ 0 & 0 & 0 & 0 & \frac{1}{2} & \frac{1}{2} & 0 & 0 \\ 0 & 0 & 0 & 0 & \frac{1}{2} & \frac{1}{2} & 0 & 0 \\ 0 & 0 & 0 & 0 & 0 & 0 & \frac{1}{2} & \frac{1}{2} \\ 0 & 0 & 0 & 0 & 0 & 0 & \frac{1}{2} & \frac{1}{2} \end{pmatrix} = \frac{1}{2}\cdot\begin{pmatrix} 1 & 1 & 0 & 0 & 0 & 0 & 0 & 0 \\ 1 & 1 & 0 & 0 & 0 & 0 & 0 & 0 \\ 0 & 0 & 1 & 1 & 0 & 0 & 0 & 0 \\ 0 & 0 & 1 & 1 & 0 & 0 & 0 & 0 \\ 0 & 0 & 0 & 0 & 1 & 1 & 0 & 0 \\ 0 & 0 & 0 & 0 & 1 & 1 & 0 & 0 \\ 0 & 0 & 0 & 0 & 0 & 0 & 1 & 1 \\ 0 & 0 & 0 & 0 & 0 & 0 & 1 & 1 \end{pmatrix}$$

Example 2. For a saturated model with two factors where the first factor has 3 levels and the second factor has 2 levels and two interaction terms. We set the number of replicates to $K = 3$.

$$\mathbf{X} = \left(\begin{array}{cccccc}
1 & 1 & 0 & 1 & 1 & 0 \\
1 & 1 & 0 & 1 & 1 & 0 \\
1 & 1 & 0 & 1 & 1 & 0 \\
\hline
1 & 1 & 0 & -1 & -1 & 0 \\
1 & 1 & 0 & -1 & -1 & 0 \\
1 & 1 & 0 & -1 & -1 & 0 \\
\hline
1 & 0 & 1 & 1 & 0 & 1 \\
1 & 0 & 1 & 1 & 0 & 1 \\
1 & 0 & 1 & 1 & 0 & 1 \\
\hline
1 & 0 & 1 & -1 & 0 & -1 \\
1 & 0 & 1 & -1 & 0 & -1 \\
1 & 0 & 1 & -1 & 0 & -1 \\
\hline
1 & -1 & -1 & 1 & -1 & -1 \\
1 & -1 & -1 & 1 & -1 & -1 \\
1 & -1 & -1 & 1 & -1 & -1 \\
\hline
1 & -1 & -1 & -1 & 1 & 1 \\
1 & -1 & -1 & -1 & 1 & 1 \\
1 & -1 & -1 & -1 & 1 & 1
\end{array}\right)$$

$$\mathbf{W} = \left(\begin{array}{cccccccccccccccccc}
w_1 & 0 & 0 & 0 & 0 & 0 & 0 & 0 & 0 & 0 & 0 & 0 & 0 & 0 & 0 & 0 & 0 & 0 \\
0 & w_1 & 0 & 0 & 0 & 0 & 0 & 0 & 0 & 0 & 0 & 0 & 0 & 0 & 0 & 0 & 0 & 0 \\
0 & 0 & w_1 & 0 & 0 & 0 & 0 & 0 & 0 & 0 & 0 & 0 & 0 & 0 & 0 & 0 & 0 & 0 \\
\cline{1-3}
0 & 0 & 0 & w_2 & 0 & 0 & 0 & 0 & 0 & 0 & 0 & 0 & 0 & 0 & 0 & 0 & 0 & 0 \\
0 & 0 & 0 & 0 & w_2 & 0 & 0 & 0 & 0 & 0 & 0 & 0 & 0 & 0 & 0 & 0 & 0 & 0 \\
0 & 0 & 0 & 0 & 0 & w_2 & 0 & 0 & 0 & 0 & 0 & 0 & 0 & 0 & 0 & 0 & 0 & 0 \\
\cline{4-6}
0 & 0 & 0 & 0 & 0 & 0 & w_3 & 0 & 0 & 0 & 0 & 0 & 0 & 0 & 0 & 0 & 0 & 0 \\
0 & 0 & 0 & 0 & 0 & 0 & 0 & w_3 & 0 & 0 & 0 & 0 & 0 & 0 & 0 & 0 & 0 & 0 \\
0 & 0 & 0 & 0 & 0 & 0 & 0 & 0 & w_3 & 0 & 0 & 0 & 0 & 0 & 0 & 0 & 0 & 0 \\
\cline{7-9}
0 & 0 & 0 & 0 & 0 & 0 & 0 & 0 & 0 & w_4 & 0 & 0 & 0 & 0 & 0 & 0 & 0 & 0 \\
0 & 0 & 0 & 0 & 0 & 0 & 0 & 0 & 0 & 0 & w_4 & 0 & 0 & 0 & 0 & 0 & 0 & 0 \\
0 & 0 & 0 & 0 & 0 & 0 & 0 & 0 & 0 & 0 & 0 & w_4 & 0 & 0 & 0 & 0 & 0 & 0 \\
\cline{10-12}
0 & 0 & 0 & 0 & 0 & 0 & 0 & 0 & 0 & 0 & 0 & 0 & w_5 & 0 & 0 & 0 & 0 & 0 \\
0 & 0 & 0 & 0 & 0 & 0 & 0 & 0 & 0 & 0 & 0 & 0 & 0 & w_5 & 0 & 0 & 0 & 0 \\
0 & 0 & 0 & 0 & 0 & 0 & 0 & 0 & 0 & 0 & 0 & 0 & 0 & 0 & w_5 & 0 & 0 & 0 \\
\cline{13-15}
0 & 0 & 0 & 0 & 0 & 0 & 0 & 0 & 0 & 0 & 0 & 0 & 0 & 0 & 0 & w_6 & 0 & 0 \\
0 & 0 & 0 & 0 & 0 & 0 & 0 & 0 & 0 & 0 & 0 & 0 & 0 & 0 & 0 & 0 & w_6 & 0 \\
0 & 0 & 0 & 0 & 0 & 0 & 0 & 0 & 0 & 0 & 0 & 0 & 0 & 0 & 0 & 0 & 0 & w_6 \\
\cline{16-18}
\end{array}\right)$$

$$\mathbf{Q} = \mathbf{W}^{(1/2)}\mathbf{X} = \begin{pmatrix}
\sqrt{w_1} & \sqrt{w_1} & 0 & \sqrt{w_1} & \sqrt{w_1} & 0 \\
\sqrt{w_1} & \sqrt{w_1} & 0 & \sqrt{w_1} & \sqrt{w_1} & 0 \\
\sqrt{w_1} & \sqrt{w_1} & 0 & \sqrt{w_1} & \sqrt{w_1} & 0 \\
\sqrt{w_2} & \sqrt{w_2} & 0 & -\sqrt{w_2} & -\sqrt{w_2} & 0 \\
\sqrt{w_2} & \sqrt{w_2} & 0 & -\sqrt{w_2} & -\sqrt{w_2} & 0 \\
\sqrt{w_2} & \sqrt{w_2} & 0 & -\sqrt{w_2} & -\sqrt{w_2} & 0 \\
\sqrt{w_3} & 0 & \sqrt{w_3} & \sqrt{w_3} & 0 & \sqrt{w_3} \\
\sqrt{w_3} & 0 & \sqrt{w_3} & \sqrt{w_3} & 0 & \sqrt{w_3} \\
\sqrt{w_3} & 0 & \sqrt{w_3} & \sqrt{w_3} & 0 & \sqrt{w_3} \\
\sqrt{w_4} & 0 & \sqrt{w_4} & -\sqrt{w_4} & 0 & -\sqrt{w_4} \\
\sqrt{w_4} & 0 & \sqrt{w_4} & -\sqrt{w_4} & 0 & -\sqrt{w_4} \\
\sqrt{w_4} & 0 & \sqrt{w_4} & -\sqrt{w_4} & 0 & -\sqrt{w_4} \\
\sqrt{w_5} & -\sqrt{w_5} & -\sqrt{w_5} & \sqrt{w_5} & -\sqrt{w_5} & -\sqrt{w_5} \\
\sqrt{w_5} & -\sqrt{w_5} & -\sqrt{w_5} & \sqrt{w_5} & -\sqrt{w_5} & -\sqrt{w_5} \\
\sqrt{w_5} & -\sqrt{w_5} & -\sqrt{w_5} & \sqrt{w_5} & -\sqrt{w_5} & -\sqrt{w_5} \\
\sqrt{w_6} & -\sqrt{w_6} & -\sqrt{w_6} & -\sqrt{w_6} & \sqrt{w_6} & \sqrt{w_6} \\
\sqrt{w_6} & -\sqrt{w_6} & -\sqrt{w_6} & -\sqrt{w_6} & \sqrt{w_6} & \sqrt{w_6} \\
\sqrt{w_6} & -\sqrt{w_6} & -\sqrt{w_6} & -\sqrt{w_6} & \sqrt{w_6} & \sqrt{w_6}
\end{pmatrix}$$

$$\hat{\mathbf{H}} = \mathbf{Q}(\mathbf{Q}'\mathbf{Q})^{-1}\mathbf{Q}' = \begin{pmatrix}
\frac{1}{3} & \frac{1}{3} & \frac{1}{3} & 0 & 0 & 0 & 0 & 0 & 0 & 0 & 0 & 0 & 0 & 0 & 0 & 0 & 0 & 0 \\
\frac{1}{3} & \frac{1}{3} & \frac{1}{3} & 0 & 0 & 0 & 0 & 0 & 0 & 0 & 0 & 0 & 0 & 0 & 0 & 0 & 0 & 0 \\
\frac{1}{3} & \frac{1}{3} & \frac{1}{3} & 0 & 0 & 0 & 0 & 0 & 0 & 0 & 0 & 0 & 0 & 0 & 0 & 0 & 0 & 0 \\
0 & 0 & 0 & \frac{1}{3} & \frac{1}{3} & \frac{1}{3} & 0 & 0 & 0 & 0 & 0 & 0 & 0 & 0 & 0 & 0 & 0 & 0 \\
0 & 0 & 0 & \frac{1}{3} & \frac{1}{3} & \frac{1}{3} & 0 & 0 & 0 & 0 & 0 & 0 & 0 & 0 & 0 & 0 & 0 & 0 \\
0 & 0 & 0 & \frac{1}{3} & \frac{1}{3} & \frac{1}{3} & 0 & 0 & 0 & 0 & 0 & 0 & 0 & 0 & 0 & 0 & 0 & 0 \\
0 & 0 & 0 & 0 & 0 & 0 & \frac{1}{3} & \frac{1}{3} & \frac{1}{3} & 0 & 0 & 0 & 0 & 0 & 0 & 0 & 0 & 0 \\
0 & 0 & 0 & 0 & 0 & 0 & \frac{1}{3} & \frac{1}{3} & \frac{1}{3} & 0 & 0 & 0 & 0 & 0 & 0 & 0 & 0 & 0 \\
0 & 0 & 0 & 0 & 0 & 0 & \frac{1}{3} & \frac{1}{3} & \frac{1}{3} & 0 & 0 & 0 & 0 & 0 & 0 & 0 & 0 & 0 \\
0 & 0 & 0 & 0 & 0 & 0 & 0 & 0 & 0 & \frac{1}{3} & \frac{1}{3} & \frac{1}{3} & 0 & 0 & 0 & 0 & 0 & 0 \\
0 & 0 & 0 & 0 & 0 & 0 & 0 & 0 & 0 & \frac{1}{3} & \frac{1}{3} & \frac{1}{3} & 0 & 0 & 0 & 0 & 0 & 0 \\
0 & 0 & 0 & 0 & 0 & 0 & 0 & 0 & 0 & \frac{1}{3} & \frac{1}{3} & \frac{1}{3} & 0 & 0 & 0 & 0 & 0 & 0 \\
0 & 0 & 0 & 0 & 0 & 0 & 0 & 0 & 0 & 0 & 0 & 0 & \frac{1}{3} & \frac{1}{3} & \frac{1}{3} & 0 & 0 & 0 \\
0 & 0 & 0 & 0 & 0 & 0 & 0 & 0 & 0 & 0 & 0 & 0 & \frac{1}{3} & \frac{1}{3} & \frac{1}{3} & 0 & 0 & 0 \\
0 & 0 & 0 & 0 & 0 & 0 & 0 & 0 & 0 & 0 & 0 & 0 & \frac{1}{3} & \frac{1}{3} & \frac{1}{3} & 0 & 0 & 0 \\
0 & 0 & 0 & 0 & 0 & 0 & 0 & 0 & 0 & 0 & 0 & 0 & 0 & 0 & 0 & \frac{1}{3} & \frac{1}{3} & \frac{1}{3} \\
0 & 0 & 0 & 0 & 0 & 0 & 0 & 0 & 0 & 0 & 0 & 0 & 0 & 0 & 0 & \frac{1}{3} & \frac{1}{3} & \frac{1}{3} \\
0 & 0 & 0 & 0 & 0 & 0 & 0 & 0 & 0 & 0 & 0 & 0 & 0 & 0 & 0 & \frac{1}{3} & \frac{1}{3} & \frac{1}{3}
\end{pmatrix}$$

We note that re-arranging the data matrix by placing together all observations per replication, we arrive a Hat matrix of blocks each with $\frac{1}{K} * \mathbf{I}_{n \times n}$, here $K = 3$ and $n = 6$.

$$\mathbf{X} = \left(\begin{array}{cccccc}
1 & 1 & 0 & 1 & 1 & 0 \\
1 & 1 & 0 & -1 & -1 & 0 \\
1 & 0 & 1 & 1 & 0 & 1 \\
1 & 0 & 1 & -1 & 0 & -1 \\
1 & -1 & -1 & 1 & -1 & -1 \\
1 & -1 & -1 & -1 & 1 & 1 \\
\hline
1 & 1 & 0 & 1 & 1 & 0 \\
1 & 1 & 0 & -1 & -1 & 0 \\
1 & 0 & 1 & 1 & 0 & 1 \\
1 & 0 & 1 & -1 & 0 & -1 \\
1 & -1 & -1 & 1 & -1 & -1 \\
1 & -1 & -1 & -1 & 1 & 1 \\
\hline
1 & 1 & 0 & 1 & 1 & 0 \\
1 & 1 & 0 & -1 & -1 & 0 \\
1 & 0 & 1 & 1 & 0 & 1 \\
1 & 0 & 1 & -1 & 0 & -1 \\
1 & -1 & -1 & 1 & -1 & -1 \\
1 & -1 & -1 & -1 & 1 & 1
\end{array}\right)$$

Similarly, rearanging the corresponding observation weights

$$\mathbf{W} = \left(\begin{array}{cccccccccccccccccc}
w_1 & 0 & 0 & 0 & 0 & 0 & 0 & 0 & 0 & 0 & 0 & 0 & 0 & 0 & 0 & 0 & 0 & 0 \\
0 & w_2 & 0 & 0 & 0 & 0 & 0 & 0 & 0 & 0 & 0 & 0 & 0 & 0 & 0 & 0 & 0 & 0 \\
0 & 0 & w_3 & 0 & 0 & 0 & 0 & 0 & 0 & 0 & 0 & 0 & 0 & 0 & 0 & 0 & 0 & 0 \\
0 & 0 & 0 & w_4 & 0 & 0 & 0 & 0 & 0 & 0 & 0 & 0 & 0 & 0 & 0 & 0 & 0 & 0 \\
0 & 0 & 0 & 0 & w_5 & 0 & 0 & 0 & 0 & 0 & 0 & 0 & 0 & 0 & 0 & 0 & 0 & 0 \\
0 & 0 & 0 & 0 & 0 & w_6 & 0 & 0 & 0 & 0 & 0 & 0 & 0 & 0 & 0 & 0 & 0 & 0 \\
\cline{1-6}
0 & 0 & 0 & 0 & 0 & 0 & w_1 & 0 & 0 & 0 & 0 & 0 & 0 & 0 & 0 & 0 & 0 & 0 \\
0 & 0 & 0 & 0 & 0 & 0 & 0 & w_2 & 0 & 0 & 0 & 0 & 0 & 0 & 0 & 0 & 0 & 0 \\
0 & 0 & 0 & 0 & 0 & 0 & 0 & 0 & w_3 & 0 & 0 & 0 & 0 & 0 & 0 & 0 & 0 & 0 \\
0 & 0 & 0 & 0 & 0 & 0 & 0 & 0 & 0 & w_4 & 0 & 0 & 0 & 0 & 0 & 0 & 0 & 0 \\
0 & 0 & 0 & 0 & 0 & 0 & 0 & 0 & 0 & 0 & w_5 & 0 & 0 & 0 & 0 & 0 & 0 & 0 \\
0 & 0 & 0 & 0 & 0 & 0 & 0 & 0 & 0 & 0 & 0 & w_6 & 0 & 0 & 0 & 0 & 0 & 0 \\
\cline{7-12}
0 & 0 & 0 & 0 & 0 & 0 & 0 & 0 & 0 & 0 & 0 & 0 & w_1 & 0 & 0 & 0 & 0 & 0 \\
0 & 0 & 0 & 0 & 0 & 0 & 0 & 0 & 0 & 0 & 0 & 0 & 0 & w_2 & 0 & 0 & 0 & 0 \\
0 & 0 & 0 & 0 & 0 & 0 & 0 & 0 & 0 & 0 & 0 & 0 & 0 & 0 & w_3 & 0 & 0 & 0 \\
0 & 0 & 0 & 0 & 0 & 0 & 0 & 0 & 0 & 0 & 0 & 0 & 0 & 0 & 0 & w_4 & 0 & 0 \\
0 & 0 & 0 & 0 & 0 & 0 & 0 & 0 & 0 & 0 & 0 & 0 & 0 & 0 & 0 & 0 & w_5 & 0 \\
0 & 0 & 0 & 0 & 0 & 0 & 0 & 0 & 0 & 0 & 0 & 0 & 0 & 0 & 0 & 0 & 0 & w_6 \\
\cline{13-18}
\end{array}\right)$$

$$
\hat{\mathbf{H}} = \left(\begin{array}{cccccc|cccccc|cccccc}
\frac{1}{3} & 0 & 0 & 0 & 0 & 0 & \frac{1}{3} & 0 & 0 & 0 & 0 & 0 & \frac{1}{3} & 0 & 0 & 0 & 0 & 0 \\
0 & \frac{1}{3} & 0 & 0 & 0 & 0 & 0 & \frac{1}{3} & 0 & 0 & 0 & 0 & 0 & \frac{1}{3} & 0 & 0 & 0 & 0 \\
0 & 0 & \frac{1}{3} & 0 & 0 & 0 & 0 & 0 & \frac{1}{3} & 0 & 0 & 0 & 0 & 0 & \frac{1}{3} & 0 & 0 & 0 \\
0 & 0 & 0 & \frac{1}{3} & 0 & 0 & 0 & 0 & 0 & \frac{1}{3} & 0 & 0 & 0 & 0 & 0 & \frac{1}{3} & 0 & 0 \\
0 & 0 & 0 & 0 & \frac{1}{3} & 0 & 0 & 0 & 0 & 0 & \frac{1}{3} & 0 & 0 & 0 & 0 & 0 & \frac{1}{3} & 0 \\
0 & 0 & 0 & 0 & 0 & \frac{1}{3} & 0 & 0 & 0 & 0 & 0 & \frac{1}{3} & 0 & 0 & 0 & 0 & 0 & \frac{1}{3} \\
\hline
\frac{1}{3} & 0 & 0 & 0 & 0 & 0 & \frac{1}{3} & 0 & 0 & 0 & 0 & 0 & \frac{1}{3} & 0 & 0 & 0 & 0 & 0 \\
0 & \frac{1}{3} & 0 & 0 & 0 & 0 & 0 & \frac{1}{3} & 0 & 0 & 0 & 0 & 0 & \frac{1}{3} & 0 & 0 & 0 & 0 \\
0 & 0 & \frac{1}{3} & 0 & 0 & 0 & 0 & 0 & \frac{1}{3} & 0 & 0 & 0 & 0 & 0 & \frac{1}{3} & 0 & 0 & 0 \\
0 & 0 & 0 & \frac{1}{3} & 0 & 0 & 0 & 0 & 0 & \frac{1}{3} & 0 & 0 & 0 & 0 & 0 & \frac{1}{3} & 0 & 0 \\
0 & 0 & 0 & 0 & \frac{1}{3} & 0 & 0 & 0 & 0 & 0 & \frac{1}{3} & 0 & 0 & 0 & 0 & 0 & \frac{1}{3} & 0 \\
0 & 0 & 0 & 0 & 0 & \frac{1}{3} & 0 & 0 & 0 & 0 & 0 & \frac{1}{3} & 0 & 0 & 0 & 0 & 0 & \frac{1}{3} \\
\hline
\frac{1}{3} & 0 & 0 & 0 & 0 & 0 & \frac{1}{3} & 0 & 0 & 0 & 0 & 0 & \frac{1}{3} & 0 & 0 & 0 & 0 & 0 \\
0 & \frac{1}{3} & 0 & 0 & 0 & 0 & 0 & \frac{1}{3} & 0 & 0 & 0 & 0 & 0 & \frac{1}{3} & 0 & 0 & 0 & 0 \\
0 & 0 & \frac{1}{3} & 0 & 0 & 0 & 0 & 0 & \frac{1}{3} & 0 & 0 & 0 & 0 & 0 & \frac{1}{3} & 0 & 0 & 0 \\
0 & 0 & 0 & \frac{1}{3} & 0 & 0 & 0 & 0 & 0 & \frac{1}{3} & 0 & 0 & 0 & 0 & 0 & \frac{1}{3} & 0 & 0 \\
0 & 0 & 0 & 0 & \frac{1}{3} & 0 & 0 & 0 & 0 & 0 & \frac{1}{3} & 0 & 0 & 0 & 0 & 0 & \frac{1}{3} & 0 \\
0 & 0 & 0 & 0 & 0 & \frac{1}{3} & 0 & 0 & 0 & 0 & 0 & \frac{1}{3} & 0 & 0 & 0 & 0 & 0 & \frac{1}{3}
\end{array}\right)
$$

In contrast, removing at least one model term or data column results in a Hat matrix where each entry is a function of the weights that depend on the response variable $(\mathbf{y})$. In such cases, the resulting $\mathbf{G}$ matrix is idempotent but not symmetric. Implying that for unsaturated models, there is no exact orthogonal decomposition of the sum of squares of the working response variable in GLMs.

Text S5: Decomposing the sum of squares of the linear predictor in balanced designs

In a balanced design, each column of the design matrix contains an equal number of observations (n). Consider a GLM that includes either all effects or a subset S of appropriately selected main and interaction effects, represented by the design matrix $\mathbf{X} = (X_0 \mid \mathbf{X}_1 \mid \ldots \mid \mathbf{X}_S)$. The corresponding linear predictor is then given by

$$\hat{\eta} = X_0\hat{\beta}_0 + \mathbf{X}_1\hat{\beta}_1 + \cdots \mathbf{X}_F\hat{\beta}_S,$$

which can also be expressed as

$$\hat{\eta} = \hat{\mathbf{H}}\hat{\mathbf{z}},$$

where $\hat{\mathbf{H}}$ is the Hat matrix:

$$\hat{\mathbf{H}} = \hat{\mathbf{W}}^{1/2}\mathbf{X}(\mathbf{X}^T\hat{\mathbf{W}}\mathbf{X})^{-1}\mathbf{X}^T\hat{\mathbf{W}}^{1/2},$$

with $\hat{\mathbf{W}} = \text{diag}(w_i), \;\; i = 1, \ldots, n.$

When an alternative link function is introduced that assigns all observation weights a constant value, say $w_i = w$, then the weight matrix becomes $\hat{\mathbf{W}} = w\mathbf{I}_{n\times n}$. Substituting this weight matrix, it follows that the Hat matrix reduces to a form analogous to that of ordinary least squares (OLS) regression (Montgomery et al., 2021):

$$\hat{\mathbf{H}} = \mathbf{X}(\mathbf{X}^T\mathbf{X})^{-1}\mathbf{X}^T,$$

In the context of a linear model, the Hat matrix $\hat{\mathbf{H}}$ can be decomposed as the sum of two or more Hat matrices (Radhakrishna Rao and Toutenburg, 1999). When the design matrix is orthogonal, this decomposition takes a particularly simple form. Specifically, for an orthogonal design matrix $\mathbf{X}$ that satisfies $\mathbf{X}_k^T\mathbf{X}_l = 0, \text{ for } k \neq l$, we have

$$\mathbf{X}^T\mathbf{X} = \text{diag}(X_0^T X_0, \mathbf{X}_1^T\mathbf{X}_1, \ldots, \mathbf{X}_S^T\mathbf{X}_S).$$

Because $\mathbf{X}^T\mathbf{X}$ is block diagonal matrix, its inverse is also block diagonal matrix and can be expressed as

$$(\mathbf{X}^T\mathbf{X})^{-1} = \text{diag}((X_0^T X_0)^{-1}, (\mathbf{X}_1^T\mathbf{X}_1)^{-1}, \ldots, (\mathbf{X}_S^T\mathbf{X}_S)^{-1}).$$

It then follows that

$$\begin{aligned}\hat{\mathbf{H}} &= X_0(X_0^T X_0)^{-1}X_0^T + \mathbf{X}_1(\mathbf{X}_1^T\mathbf{X}_1)^{-1}\mathbf{X}_1^T + \cdots + \mathbf{X}_S(\mathbf{X}_S^T\mathbf{X}_S)^{-1}\mathbf{X}_S^T \\ &= \mathbf{H_0} + \mathbf{H_1} + \cdots + \mathbf{H_S},\end{aligned}$$

where $\mathbf{H_j} = \mathbf{X}_k(\mathbf{X}_k^T\mathbf{X}_k)^{-1}\mathbf{X}_k^T, \ \ k = 0, 1, \ldots, S$, are the Hat matrices corresponding to each $\mathbf{X}_k$. These matrices are mutually orthogonal, satisfying, $\mathbf{H_k}\mathbf{H_l} = \mathbf{0}, \text{ for } k \neq l$.

Using the decomposition of $\hat{\mathbf{H}}$ as the sum of orthogonal Hat matrices, the linear predictor is expressed as

$$\hat{\eta} = \mathbf{H_0}\hat{\mathbf{z}} + \mathbf{H_1}\hat{\mathbf{z}} + \cdots + \mathbf{H_S}\hat{\mathbf{z}},$$

The orthogonal decomposition of the sum of squares of the linear predictor follows as

$$\begin{aligned}\|\hat{\eta}\|^2 &= \|\mathbf{H_0}\hat{\mathbf{z}} + \mathbf{H_1}\hat{\mathbf{z}} + \cdots + \mathbf{H_S}\hat{\mathbf{z}}\|^2 \\ &= \|\mathbf{H_0}\hat{\mathbf{z}}\|^2 + \|\mathbf{H_1}\hat{\mathbf{z}}\|^2 + \cdots + \|\mathbf{H_S}\hat{\mathbf{z}}\|^2 \\ &= \|X_0\hat{\beta}_0\|^2 + \|\mathbf{X}_1\hat{\beta}_1\|^2 + \|\mathbf{X}_2\hat{\beta}_2\|^2 + \cdots + \|\mathbf{X}_S\hat{\beta}_S\|^2,\end{aligned}$$

where $\mathbf{H_k}\hat{\mathbf{z}} = \mathbf{X}_k(\mathbf{X}_k^T\mathbf{X}_k)^{-1}\mathbf{X}_k^T\hat{\mathbf{z}} = \mathbf{X}_k\hat{\beta}_k$, with $\hat{\beta}_k = (\mathbf{X}_k^T\mathbf{X}_k)^{-1}\mathbf{X}_k^T\hat{\mathbf{z}}, \ \ k = 0, 1, \ldots, S$, resulting from the weights being equal and the orthogonal structure of the design matrix.

2 SUPPLEMENTARY TABLES AND FIGURES

2.1 Tables

Table S1. Similarity between the representations in Generalized Linear Models (GLMs) and Linear Models (LMs).

GLMs	LMs
i. working response values $\hat{\mathbf{z}}$	i. observations $\mathbf{y}$
ii. estimated linear predictor $\hat{\eta}$	ii. fitted values $\hat{\mathbf{y}}$

iii. working residuals $\mathbf{r}^w = \hat{\mathbf{z}} - \hat{\eta}$

iv. the scaled fitted values $\hat{\eta}^*$ are the orthogonal projections of the scaled-adjusted responses $\hat{\mathbf{z}}^*$ on the linear space spanned by the columns of $\hat{\mathbf{W}}^{1/2}\mathbf{X}$, with hat matrix in GLMs which is idempotent and symmetric

$$\hat{\mathbf{H}} = \hat{\mathbf{W}}^{1/2}\mathbf{X}(\mathbf{X}^T\hat{\mathbf{W}}\mathbf{X})^{-1}\mathbf{X}^T\hat{\mathbf{W}}^{1/2}$$

v. $\hat{\eta}^* = \hat{\mathbf{H}}\hat{\mathbf{z}}^*$

vi. the scaled working residuals or adjusted residuals $(\mathbf{r}^{w*})$ are equal to the Pearson residuals $(\mathbf{r}^p)$

$$\mathbf{r}^p = \hat{\mathbf{z}}^* - \hat{\eta}^* = \hat{\mathbf{W}}^{1/2}\mathbf{r}^W = \frac{\mathbf{y} - \hat{\mu}}{\sqrt{(V(\hat{\mu})}}$$

vii. $\mathbf{r}^p = \mathbf{r}^{w*} = (\mathbf{I} - \hat{\mathbf{H}})\hat{\mathbf{z}}^*$

viii. the adjusted residuals $\mathbf{r}^{w*}$ are orthogonal to the adjusted fitted values $\hat{\eta}^*$

$$\hat{\eta}^{*T}\mathbf{r}^{w*} = \hat{\mathbf{z}}^{*T}\hat{\mathbf{H}}\left(\mathbf{I} - \hat{\mathbf{H}}\right)\hat{\mathbf{z}}^* = 0$$

ix. the adjusted residuals provide an exact orthogonal decomposition of the sum of squares of the adjusted response

$$\hat{\mathbf{z}}^{*T}\hat{\mathbf{z}}^* = \hat{\eta}^{*T}\hat{\eta}^* + \mathbf{r}^{w*T}\mathbf{r}^{w*}$$

x. partial residuals Cook and Croos-Dabrera (1998) pr_j^w for the jth variable X_j based on working residuals $\mathbf{r}^w$ is given by

$$pr_j^w = \mathbf{r}^w + X_j\hat{\beta}_j$$

Similarly, adjusted partial residualsHines and Carter (1993) pr_j^{w*} for the jth variable X_j based on Pearson residuals $\mathbf{r}^p$ is given by

$$pr_j^{w*} = \mathbf{r}^p + \hat{\mathbf{W}}^{1/2}X_j\hat{\beta}_j$$

iii. ordinary residuals $\mathbf{r} = \mathbf{y} - \hat{\mathbf{y}}$

iv. the fitted values $\hat{\mathbf{y}}$ are the orthogonal projections of the responses $\mathbf{y}$ on the linear space spanned by the columns of $\mathbf{X}$, with hat matrix in LMs which is idempotent and symmetric

$$\mathbf{H} = \mathbf{X}(\mathbf{X}^T\mathbf{X})^{-1}\mathbf{X}^T$$

v. $\hat{\mathbf{y}} = \mathbf{H}\mathbf{y}$

vi. the scaled residuals $(\mathbf{r})$ are the Pearson residuals $(\mathbf{r}^p)$

$$\mathbf{r}^p = \frac{\mathbf{y} - \hat{\mathbf{y}}}{\hat{\sigma}}$$

vii. $r = (\mathbf{I} - \mathbf{H})\mathbf{y}$

viii. the residuals $\mathbf{r}$ are orthogonal to the fitted values $\hat{\mathbf{y}}$

$$\hat{\mathbf{y}}^T\mathbf{r} = \mathbf{y}^T H\left[\mathbf{I} - \mathbf{H}\right]\mathbf{y} = 0$$

ix. the residuals provide an exact orthogonal decomposition of the sum of squares of the response

$$\mathbf{y}^T\mathbf{y} = \hat{\mathbf{y}}^T\hat{\mathbf{y}} + \mathbf{r}^T\mathbf{r}$$

x. partial residuals pr_j for the jth variable X_j

$$pr_j = \mathbf{r} + X_j\hat{\beta}_j$$

2.2 Figures

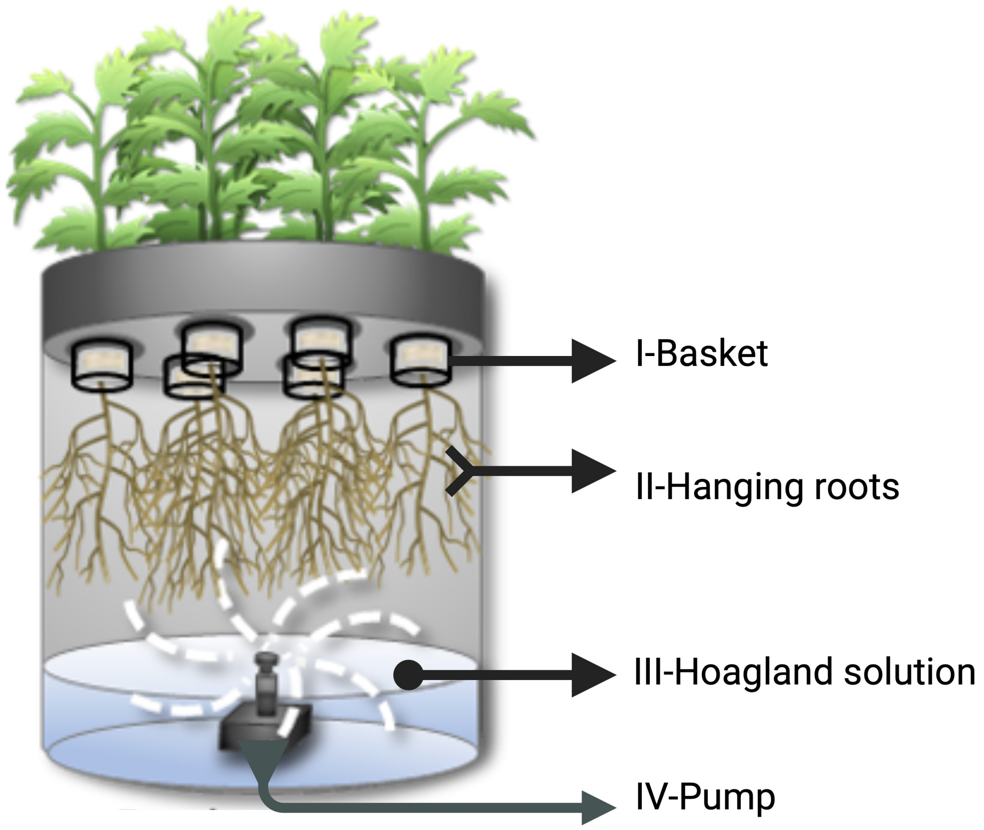

Figure S1. Schematic representation of the aeroponics system used in this study. Tomato plants are grown in small baskets filled with greenhouse soil(I), with their roots suspended in the air (II) and periodically sprayed with ¼ Hoagland solution (III) using a pump (IV). The remaining Hoagland solution was used for microbiome analysis.

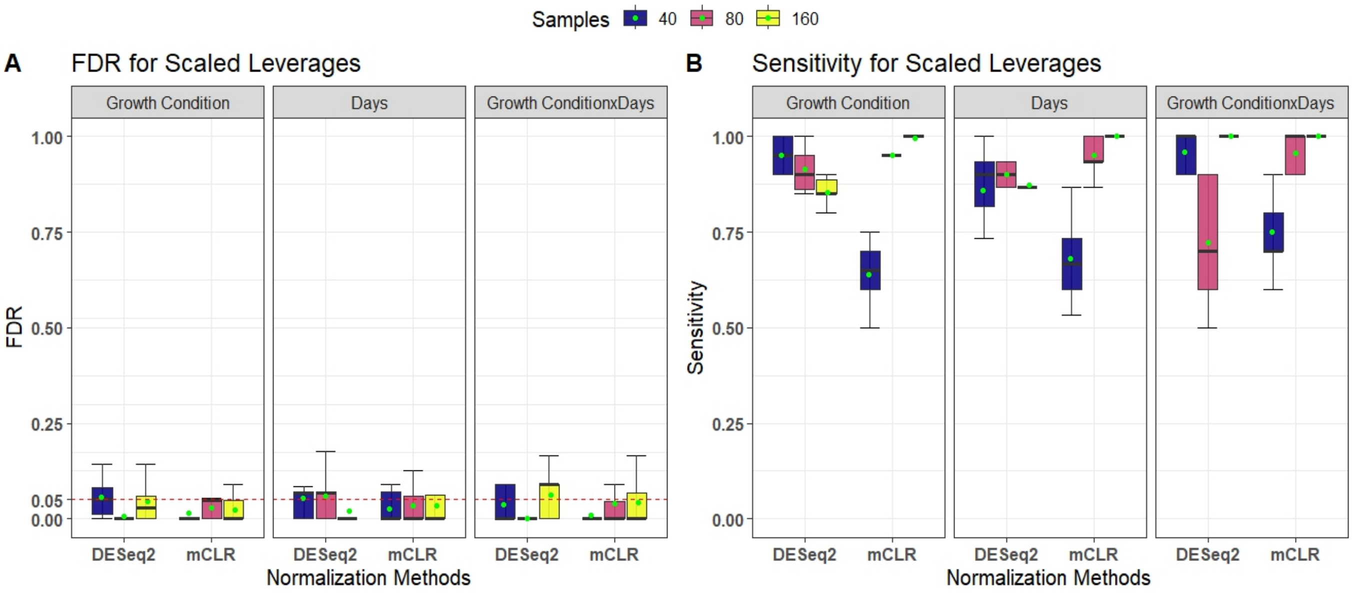

Figure S2. Effect level performance measures (FDR, and statistical power (sensitivity)) for GLM–ASCA using DESeq2 (poscounts) and mCLR normalizations evaluated using simulated data derived from a template plant microbiome dataset. Boxplots are colored by total sample size. A. Using both normalizations GLM-ASCA with scaled leverages based permutation feature selection demonstrated mean (green colored dots) FDR close to the nominal 5% level. B. GLM-ASCA with DESeq2 (poscounts) achieved high power in small samples scenario with while GLM-ASCA with mCLR achieved high power in large sample scenarios. For the binary effect variable (growth condition), the power of GLM–ASCA with DESeq2 (poscounts) exhibited a slight decrease as the sample size increased.

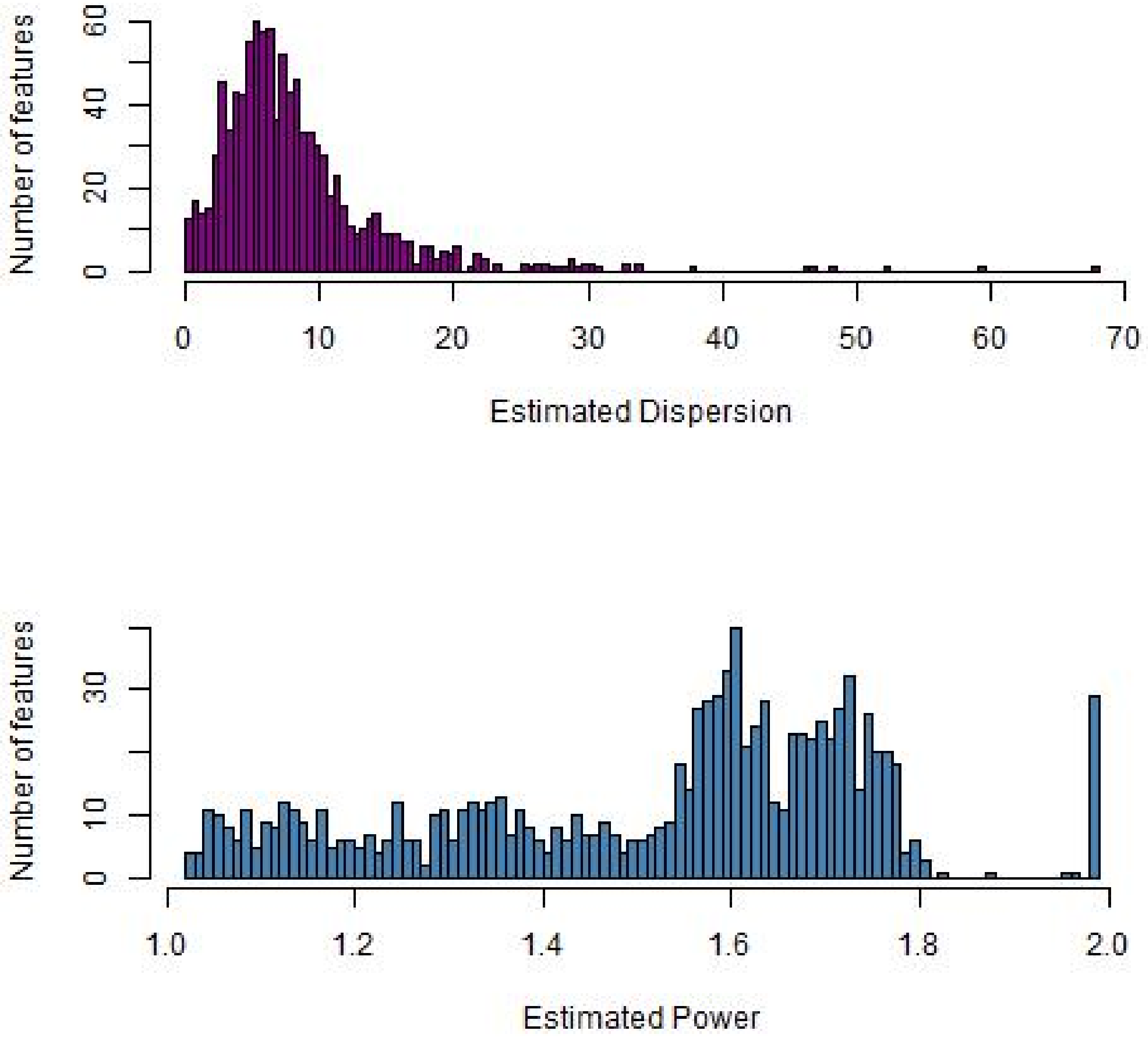

Figure S3. Power and dispersion parameter estimates of taxa using Tweedie model on plant microbial abundance data

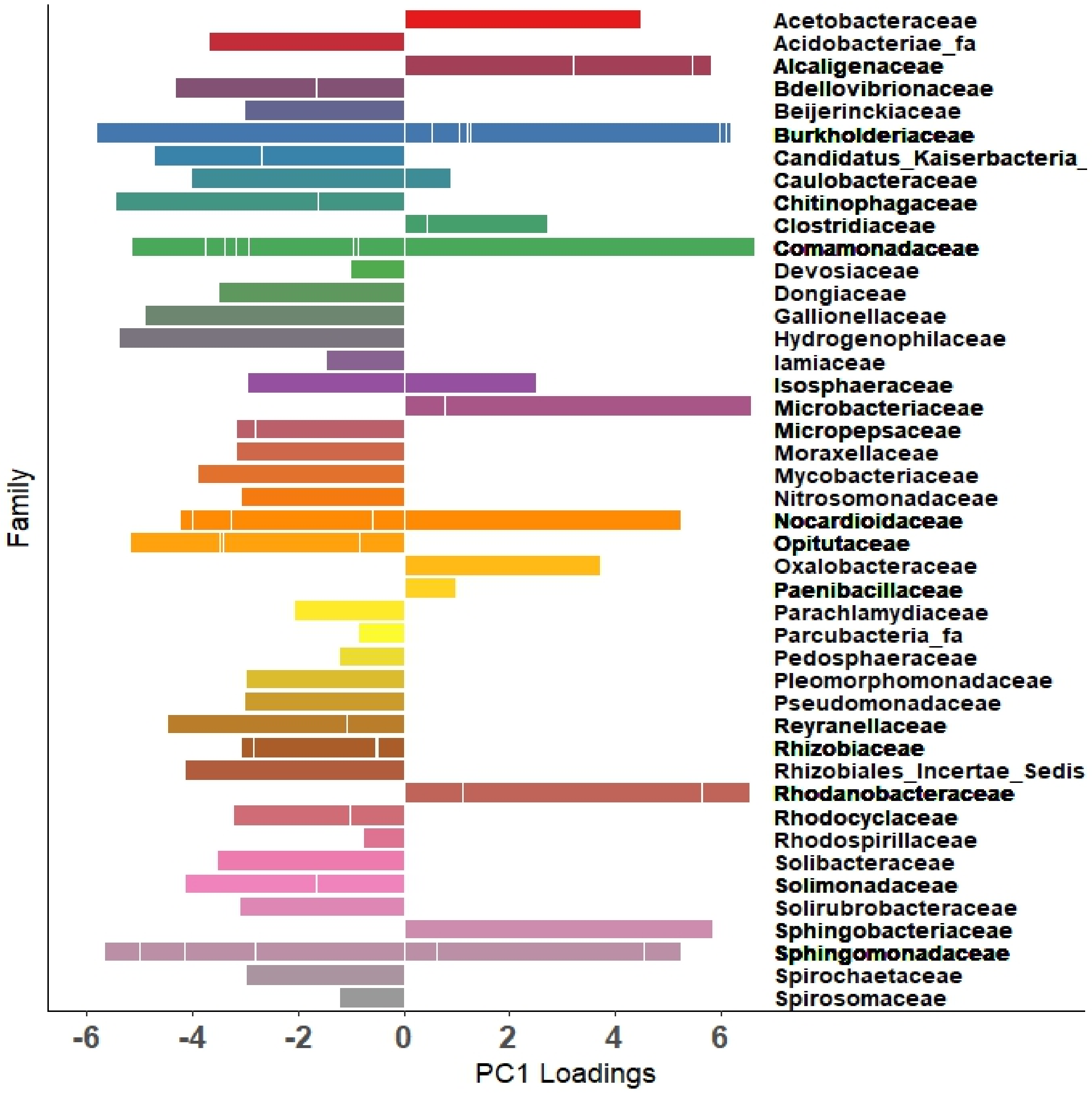

Figure S4. First principal component scores (A) and loadings (B) visualizing the effect of nitrogen starvation over time (growth condition + growth condition x Days) on microbial abundance at in the Hoagland solution with full **Family** names.

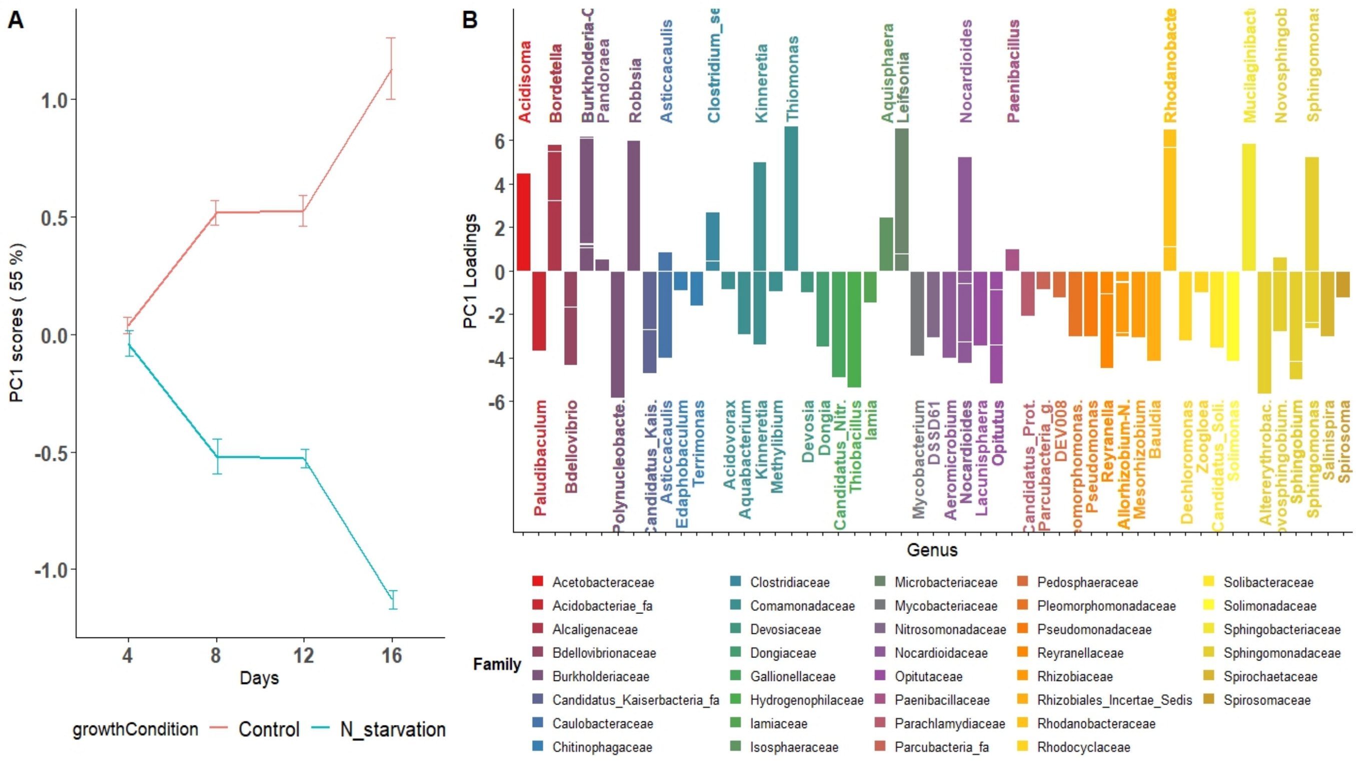

Figure S5. First principal component scores (A) and loadings (B) visualizing the effect of nitrogen starvation over time (growth condition + growth condition x Days) on microbial abundance at **Genus** level in the Hoagland solution.

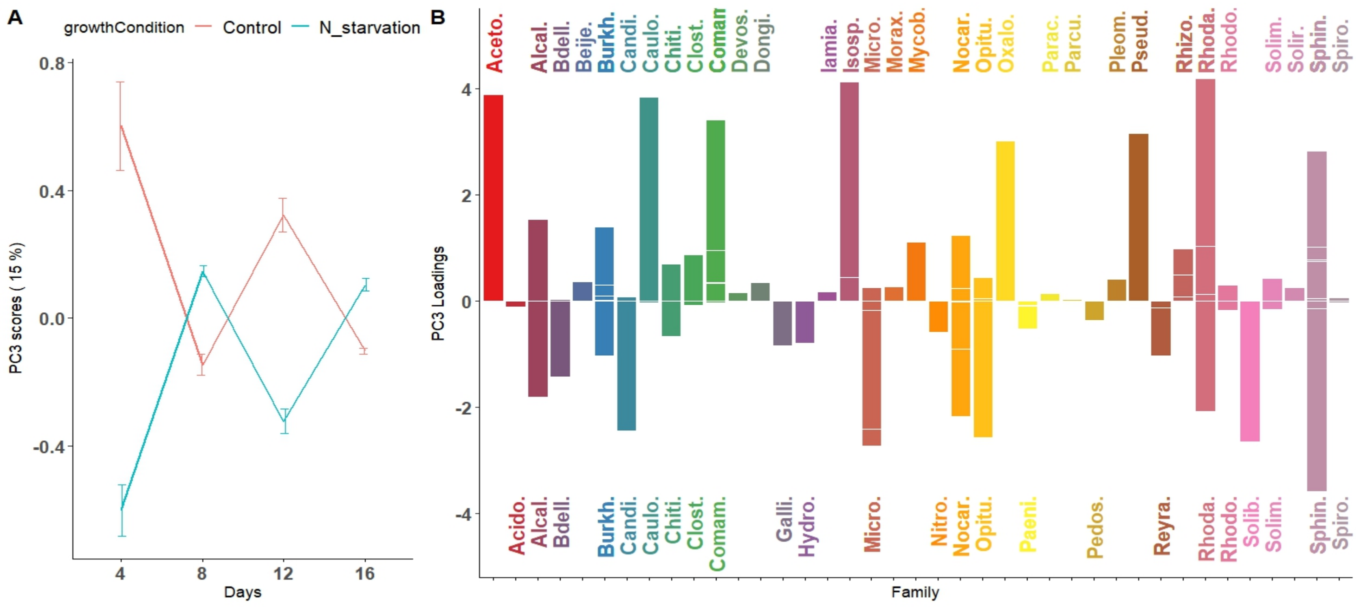

Figure S6. Third principal component scores (A) and loadings (B) visualizing the effect of nitrogen starvation over time (growth condition + growth condition x Days) on microbial abundance in the Hoagland solution.

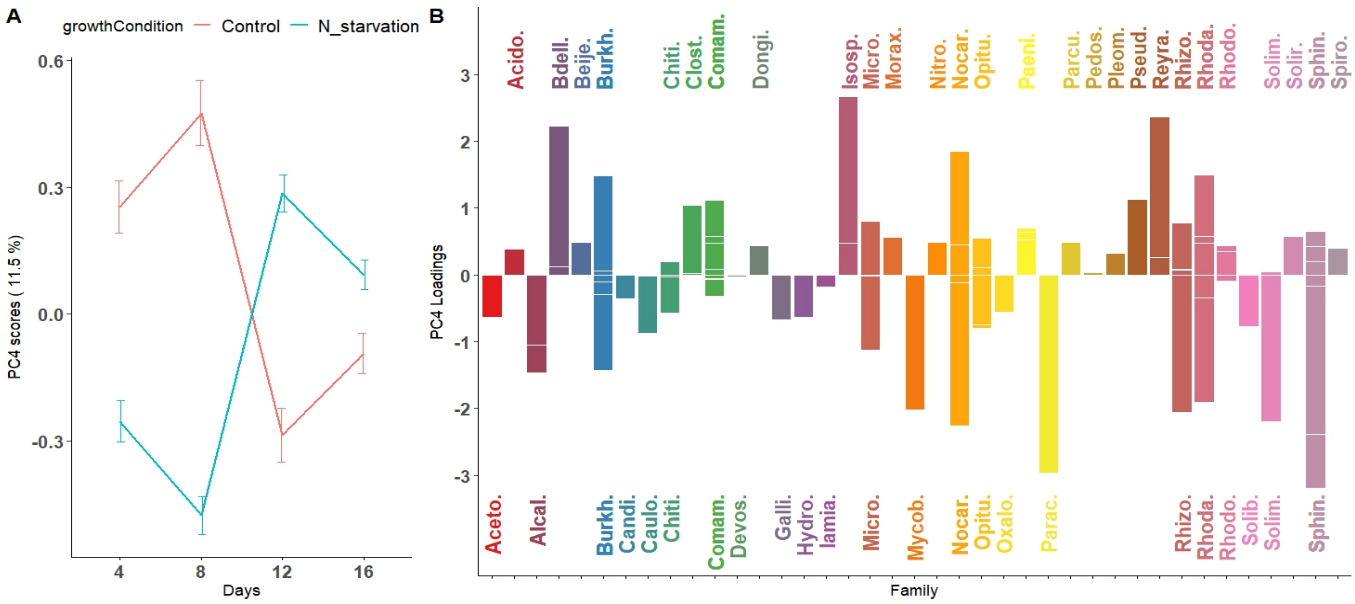

Figure S7. Fourth principal component scores (A) and loadings (B) visualizing the effect of nitrogen starvation over time (growth condition + growth condition x Days) on microbial abundance in the Hoagland solution.

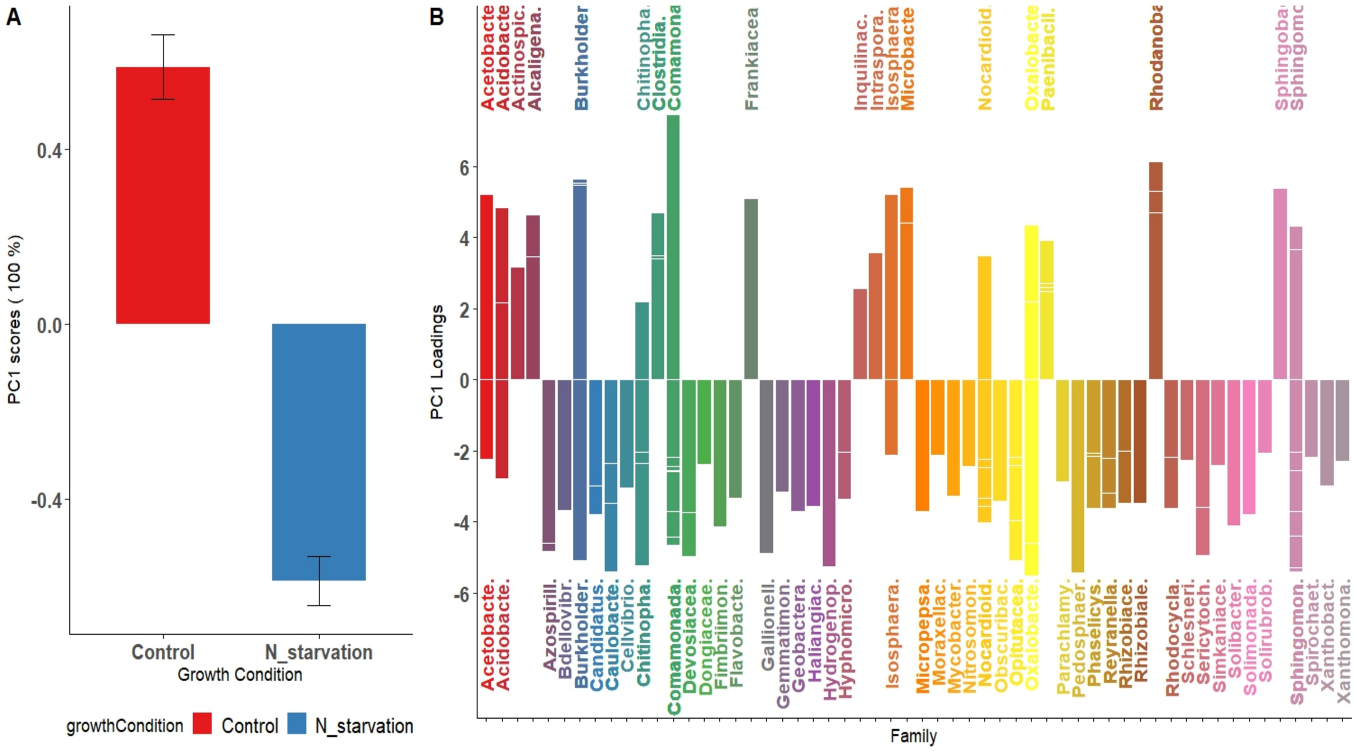

Figure S8. First principal component scores (A) and loadings (B) visualizing the marginal effect of growth condition on microbial abundance in the Hoagland solution.

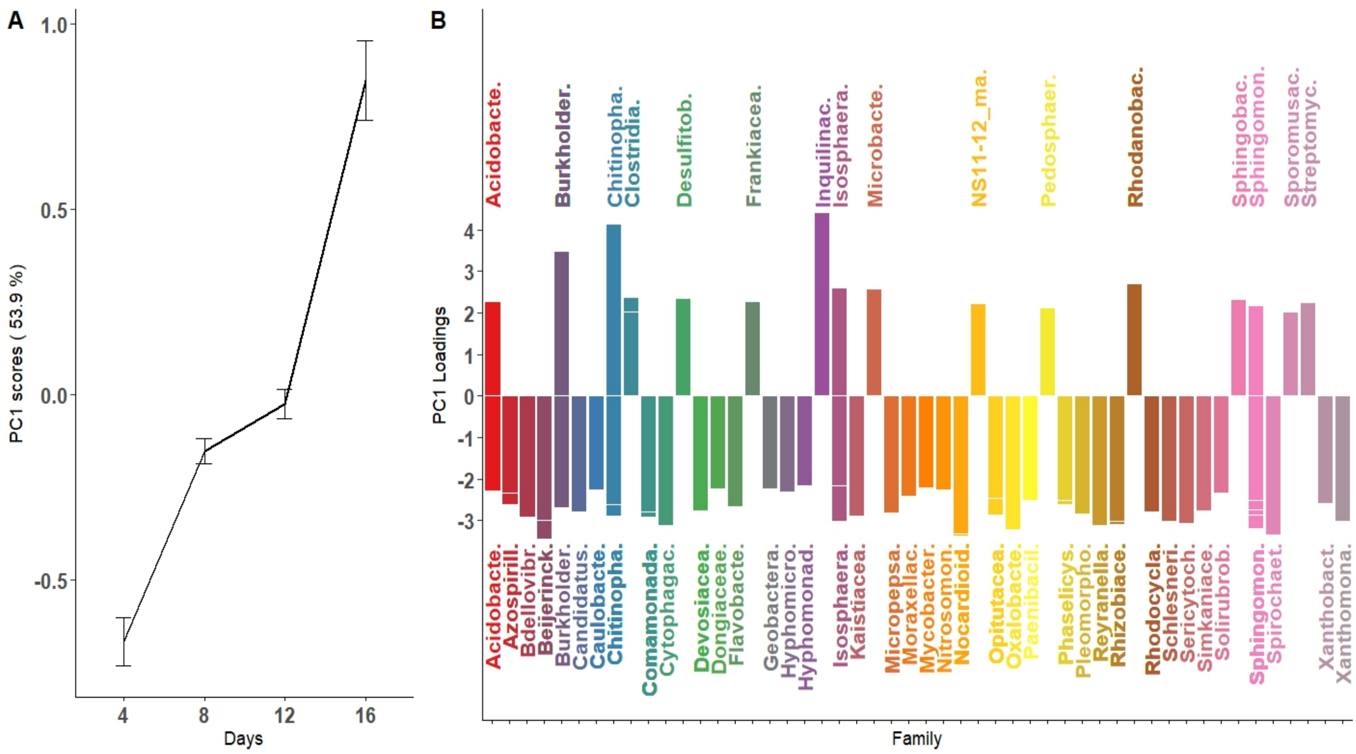

Figure S9. First principal component scores (A) and loadings (B) visualizing the marginal effect of time (in days) on microbial abundance in the Hoagland solution.

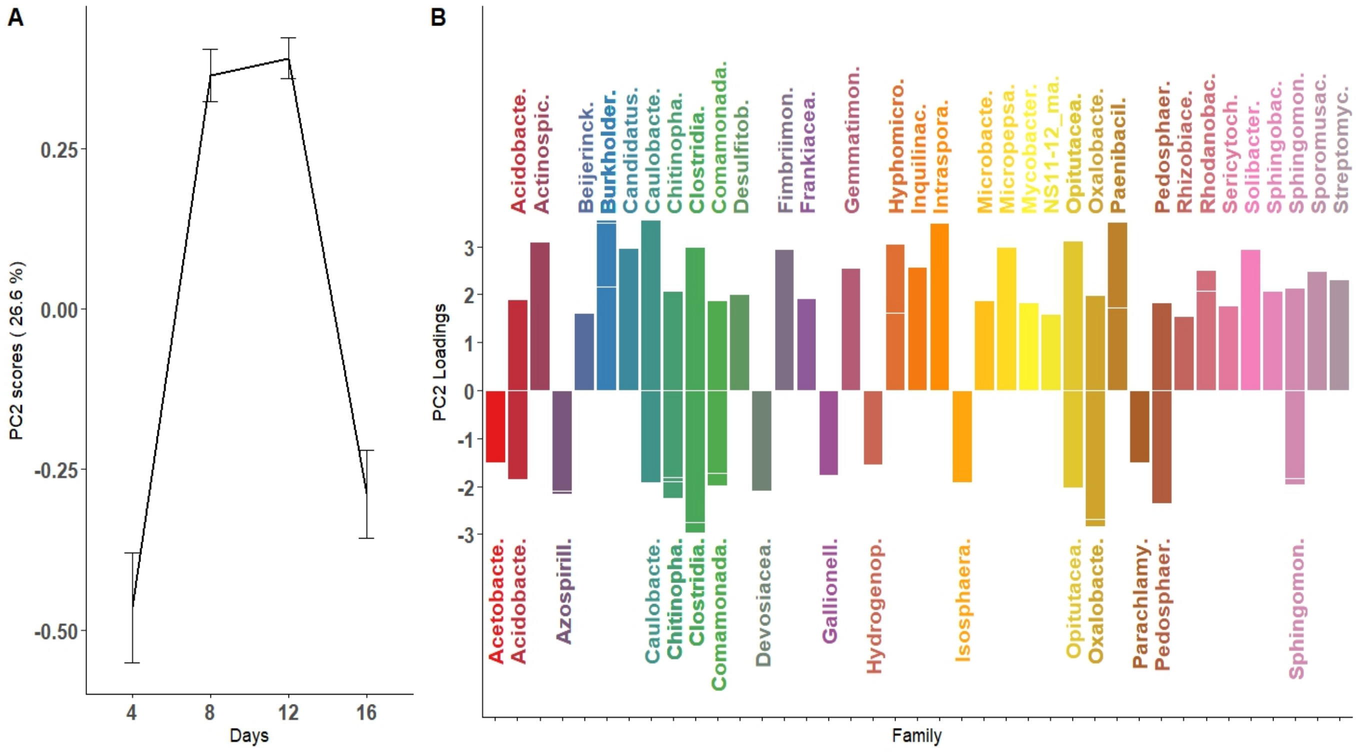

Figure S10. Second principal component scores (A) and loadings (B) visualizing the marginal effect of time (in days) on microbial abundance in the Hoagland solution.

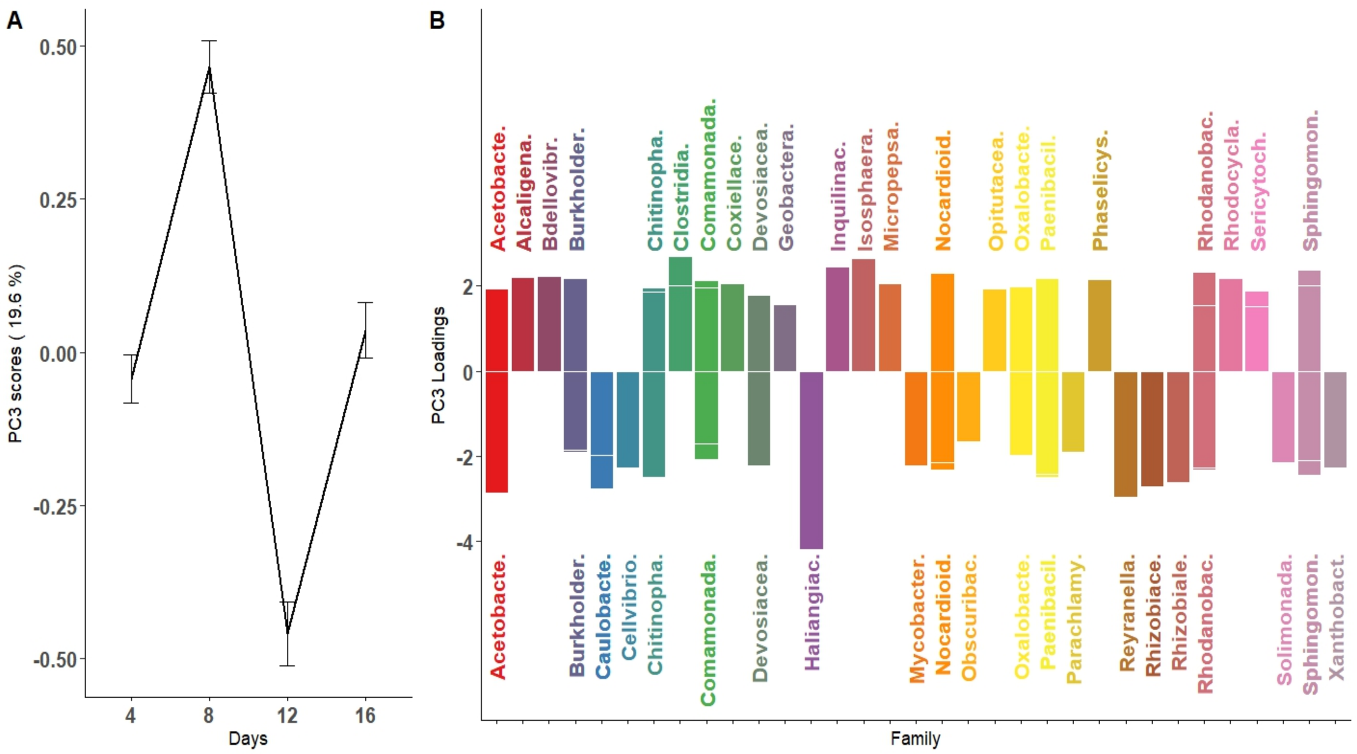

Figure S11. Third principal component scores (A) and loadings (B) visualizing the marginal effect of time (in days) on microbial abundance in the Hoagland solution.

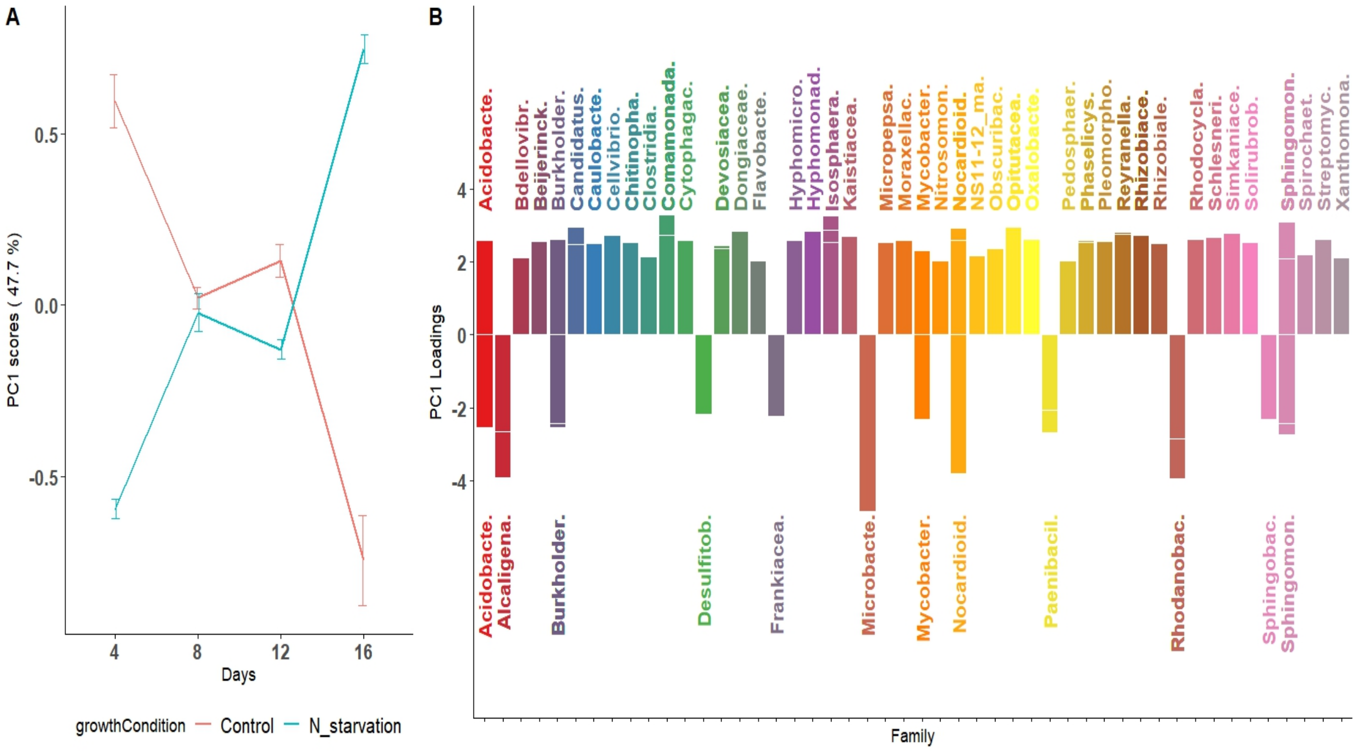

Figure S12. First principal component scores (A) and loadings (B) visualizing the interaction effect of growth condition and time (in days) on microbial abundance in the Hoagland solution.

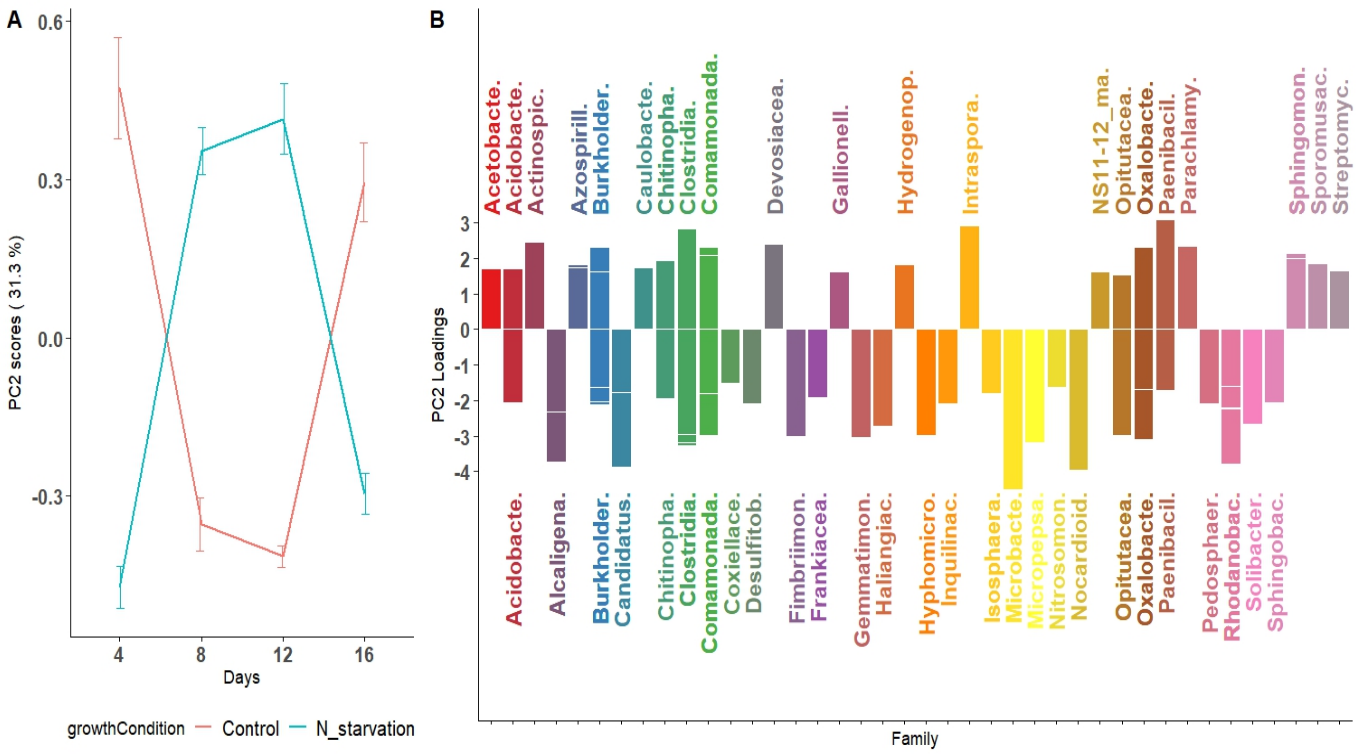

Figure S13. Second principal component scores (A) and loadings (B) visualizing the interaction effect of growth condition and time (in days) on microbial abundance in the Hoagland solution.

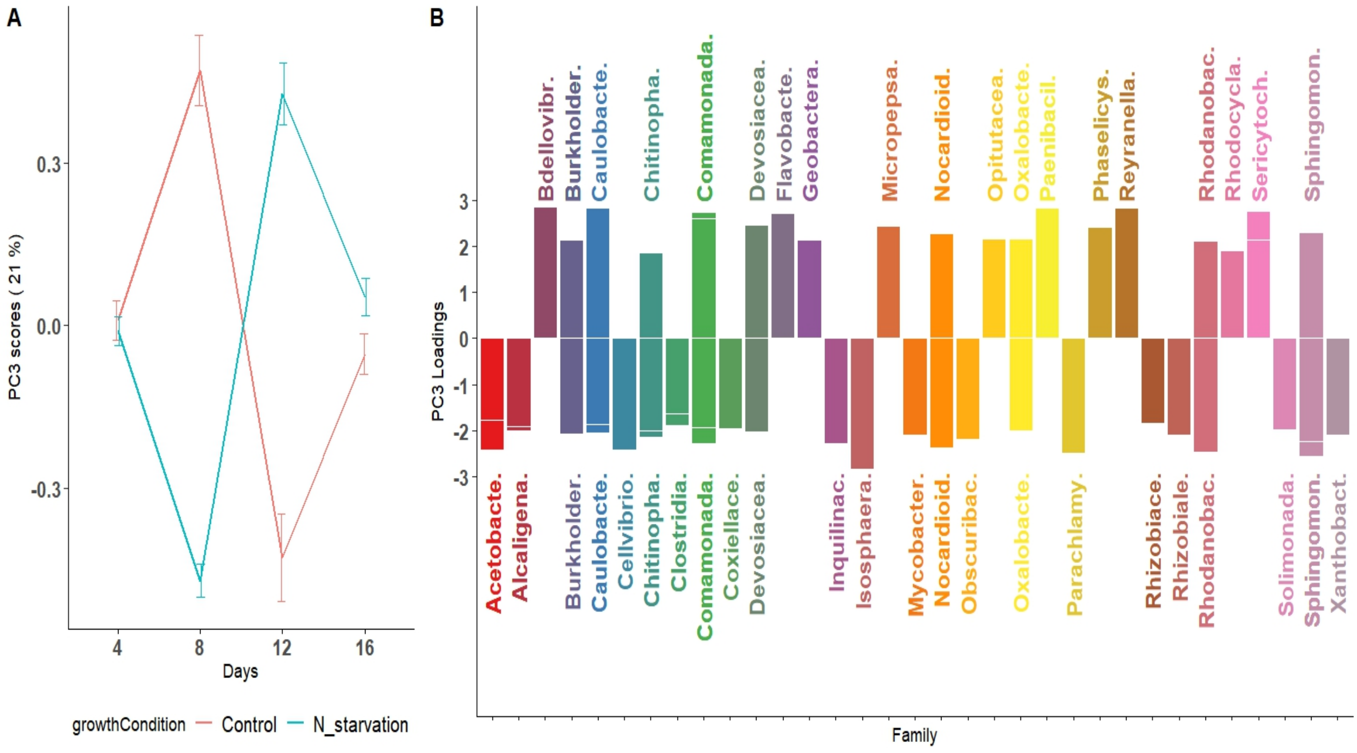

Figure S14. Third principal component scores (A) and loadings (B) visualizing the interaction effect of growth condition and time (in days) on microbial abundance in the Hoagland solution.

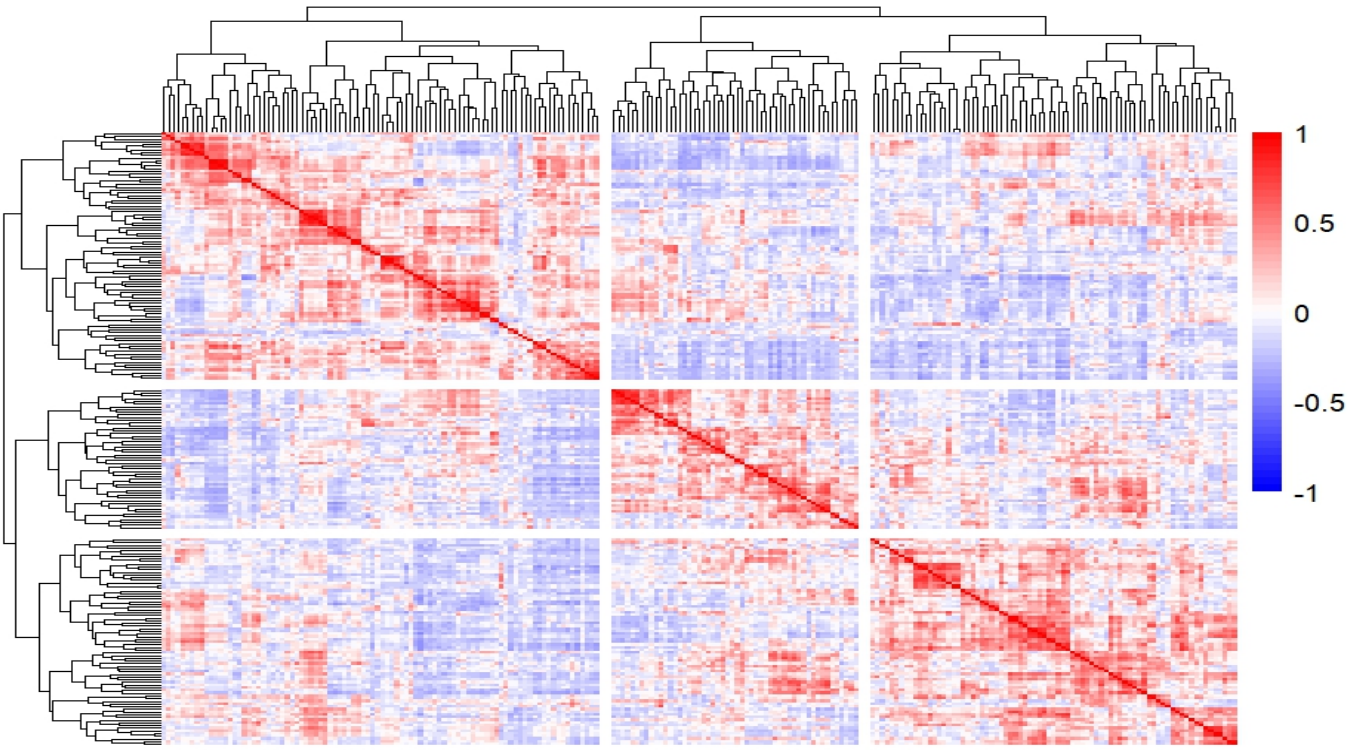

Figure S15. Correlation heatmap of 206 features (ASVs) in the filtered template plant microbiome dataset. The color scale ranges from –1 (blue, strong negative correlation) to 1 (red, strong positive correlation). The plot reveals predominantly low pairwise correlations, indicating weak dependence among the majority of features.

REFERENCES

Cook, R. D. and Croos-Dabrera, R. (1998). Partial residual plots in generalized linear models. *Journal of the American Statistical Association* 93, 730–739

Hines, R. O. and Carter, E. (1993). Improved added variable and partial residual plots for the detection of influential observations in generalized linear models. *Journal of the Royal Statistical Society, Series C (Applied Statistics)* 42, 3–16

Lovison, G. (2014). A note on adjusted responses, fitted values and residuals in Generalized Linear Models. *Statistical Modelling* 14, 337–359

McCullagh, P. and Nelder, J. A. (1989). Binary data. In *Generalized linear models* (Springer). 98–148

Montgomery, D. C., Peck, E. A., and Vining, G. G. (2021). *Introduction to linear regression analysis* (John Wiley & Sons)

Orenti, A., Marano, G., Boracchi, P., and Marubini, E. (2012). Pinpointing outliers in experimental data: the Hat matrix in Anova for fixed and mixed effects models. *Italian Journal of Public Health* 9

Radhakrishna Rao, C. and Toutenburg, H. (1999). *Linear models: least squares and alternatives* (Springer)
